# Inactivated *Rothia nasimurium* promotes a persistent antiviral immune status in porcine alveolar macrophages

**DOI:** 10.3389/fimmu.2025.1584092

**Published:** 2025-06-03

**Authors:** Aida Tort-Miró, Uxía Alonso, Beatriz Martín-Mur, Jordana Muñoz-Basagoiti, Yan Zeng, David Marín-Moraleda, Enrique Ezcurra, Sergio Montaner-Tarbes, María J. Navas, Marta Muñoz, Paula Monleón, Judith González-Oliver, Esmeralda Cano, Carles Vilalta, Marc Caballé, Lourdes Criado, Karl Kochanowski, Francesc Accensi, Virginia Aragón, Florencia Correa-Fiz, Anna Esteve-Codina, Fernando Rodríguez, Jordi Argilaguet

**Affiliations:** ^1^ IRTA, Animal Health, Centre de Recerca en Sanitat Animal (CReSA), Campus de la Universitat Autònoma de Barcelona (UAB), Bellaterra, Catalonia, Spain; ^2^ Unitat mixta d’investigació IRTA-UAB en Sanitat Animal, Centre de Recerca en Sanitat Animal (CReSA), Campus de la Universitat Autònoma de Barcelona (UAB), Bellaterra, Catalonia, Spain; ^3^ WOAH Collaborating Centre for the Research and Control of Emerging and Re-Emerging Swine Diseases in Europe (IRTA-CReSA), Bellaterra, Spain; ^4^ WPI Immunology Frontier Research Center, Osaka University, Suita, Japan; ^5^ Centro Nacional de Análisis Genómico (CNAG), Barcelona, Spain; ^6^ Universitat de Barcelona (UB), Barcelona, Spain; ^7^ College of Veterinary Medicine, Jilin Agricultural University, Changchun, China; ^8^ IRTA, Valorization Office, Torre Marimon, Caldes de Montbui, Catalonia, Spain; ^9^ Departament de Sanitat i Anatomia Animals, Facultat de Veterinària, Campus de la Universitat Autònoma de Barcelona (UAB), Bellaterra, Barcelona, Spain

**Keywords:** *Rothia nasimurium*, immunostimulant, adjuvants, antivirals, innate immune memory, porcine alveolar macrophages

## Abstract

Globalization has increased the incidence of infectious diseases in livestock, further aggravated by the reduction of antibiotic usage. To minimize the resulting economic consequences to the meat production industry, as well as the risk of zoonotic events, the use of immunostimulants has emerged as a potential strategy to enhance animal resilience to diseases. In particular, the capability of bacterial-based immunostimulants to modulate innate immune cells functionality makes them cost-effective candidates as vaccine adjuvants, antimicrobials, or preventive immunostimulators inducing long-term innate immune memory in livestock. However, further research is required to identify novel bacterial strains with immunostimulatory properties. Here we characterized *in vitro* the immunostimulatory properties of *Rothia nasimurium* isolated from warthog fecal microbiota. Stimulation with heat-inactivated *Rothia* induced cytokine production by porcine immune cells, and a robust innate immune transcriptomic signature in porcine alveolar macrophages. Interestingly, the bacteria induced inflammasome activation and IL-1β production, thus confirming its pro-inflammatory properties, and suggesting its potential as vaccine adjuvant. Importantly, this immunostimulatory status functionally resulted in an antimicrobial state, enhancing the phagocytic capability of alveolar macrophages, and hampering the replication levels of two major porcine viral pathogens: the porcine reproductive and respiratory syndrome virus (PRRSV) and the African swine fever virus (ASFV). Moreover, macrophages showed an enhanced cytokine response upon ASFV infection several days after heat-inactivated *Rothia* stimulation, suggesting the induction of an innate immune memory phenotype. This nonspecific response resulted in a significant reduction of ASFV replication kinetics, demonstrating the capacity of the bacteria to induce a more resistant state in macrophages against a virus infection. Altogether, these results demonstrate the immunostimulatory capability of heat-inactivated *R. nasimurium* in porcine macrophages, showing potential to enhance animal resilience to diseases through the modulation of innate immune cells responsiveness to infections.

## Introduction

Infectious diseases are a constant threat to livestock animals, affecting their health and welfare, causing important economic consequences to the meat production industry, and in some cases increasing the risk of zoonotic events (1). In particular, the global pork market, an important value-added activity, is severely affected by swine diseases such as porcine reproductive and respiratory syndrome (PRRS), African swine fever (ASF), porcine epidemic diarrhea, swine influenza virus, as well as *Glaesserella parasuis*, and *Streptococcus suis* infections (2). This situation is aggravated by the strict regulation implemented to antibiotic use in livestock, in order to reduce the appearance and spread of bacterial antimicrobial resistance in both animals and humans (3, 4). To address this issue, there is a dire need for the development of strategies to enhance animal resilience to diseases. These include the generation of disease-specific vaccines or their improvement with novel adjuvants, and the use of nonspecific components such as immunomodulatory agents, bacteriophages and their lysins, as well as antimicrobial peptides (AMPs) (5–8). In particular, the use of bacterial-based immunostimulants, or their derivatives, have garnered significant attention due to their direct activation of the innate immune system (9–12).

To be effective, an immunostimulant needs to interact with pattern recognition receptors (PRRs), mimicking the recognition of pathogens by the innate immune system and triggering an inflammatory response. PRRs are mainly expressed in innate immune cells such as monocytes, macrophages, dendritic cells, and neutrophils, as well as by epithelial cells (13, 14). In pigs, as in humans and other species, there are several types of PRRs, such as the toll-like receptors (TLRs), the cyclic GMP-AMP synthase-stimulator of interferon genes (cGAS-STING), the C-type lectin receptors (CLR), and the NOD-like receptors (NLRs) (15). Upon PRRs activation, the molecules involved in signal transduction converge in several common signaling pathways, including the ones mediated by the nuclear factor kappa-light-chain-enhancer of activated B cells (NF-kB) or the interferon regulatory factor 3 (IRF-3) transcription factors, the mitogen-activated protein kinase (MAPK), and the inflammasome complex (13). Upon the triggering of these pathways, activated cells functionally respond by promoting and anti-microbial state based on the expression of antiviral genes and the secretion of cytokines and chemokines, resulting in the consequent signaling and coordination with other cells from the innate and adaptive immune system (16). In some circumstances, stimulated innate immune cells can also differentiate to acquire an innate immune memory phenotype, based on a long-term, faster and enhanced response to secondary homologous or heterologous challenges (17), thus increasing the host’s resilience to infections.

Some bacterial components are highly effective in activating innate immune pathways due to their easy recognition by the PRRs (18). Indeed, there is an increasing interest on the potential use of the natural immunomodulatory properties of bacterial-based products to enhance livestock health. Applications vary from vaccine adjuvants (19), to probiotics and postbiotics (20–22). Interestingly, several studies in humans and mice have demonstrated that the modulation of the immune system through the treatment with heat-inactivated bacteria confers a variety of advantageous outcomes, from prevention of disease-associated inflammatory responses (23), to the induction of innate immune memory and a consequent heterologous protection against infections (24, 25). Importantly, inactivated bacteria fulfil some specific criteria from the veterinary pharmaceutical industry, such as easy and cost-effective manufacturing, as well as a good safety profile (26). Indeed, some inactivated bacteria have demonstrated their beneficial effects as vaccines, or their capability to unspecifically improve animal productivity and disease resilience (27–29). It is worth noting that some inactivated bacteria are already used in the veterinary market, such as the treatment with *Mycobacterium* cell wall fractions or with inactivated *Mycobacterium bovis* or *Propionibacterium* sp., having a positive impact in animal health and production (30–39). Thus, the identification of novel inactivated bacteria with immunostimulatory properties is a promising strategy to enhance resilience to diseases in farm animals.

*Rothia* spp. are Gram-positive bacteria represented by several species, some of them present in the oral cavity and respiratory tract of mammals. Although a few strains can cause opportunistic infections, most of the species are part of the commensal microbiota (40, 41). Some *Rothia* spp. have shown immunomodulatory properties, varying from anti-inflammatory to immunostimulatory effects (42, 43). Similarly, we have recently isolated a *Rothia* strain from warthog (*Phacochoerus africanus*) fecal microbiota, which showed potential as immunostimulant in a preliminary study through the induction of IFN-gamma (IFNγ) in porcine gut-associated lymphoid tissue (GALT) upon *in vitro* stimulation (44). In the present study, we aimed to better characterize the immunostimulatory capacity of this *Rothia* strain. Full genome sequencing showed that this warthog strain belongs to the *Rothia nasimurium* species. *In vitro* studies using porcine immune cells demonstrated its potent immunostimulatory properties. Importantly, heat-inactivated *R. nasimurium* broadly activated innate immune responses in porcine alveolar macrophages (PAMs), including the induction of interleukin-1 beta (IL-1β) production through inflammasome activation, thus suggesting its potential as vaccine adjuvant. Moreover, the treatment of PAMs with inactivated *R. nasimurium* reduced the replication capability of two of the most relevant swine pathogens: the porcine reproductive and respiratory syndrome virus (PRRSV) and the African swine fever virus (ASFV). Importantly, this lower susceptibility to infection was maintained over time, and concomitant with an increase of the cytokine production by infected cells, suggesting the acquisition of innate immune memory in treated-PAMs. These results indicate that the treatment of pigs with this novel inactivated *R. nasimurium* might boost the host’s immune system, thus representing a novel potential immunostimulant for livestock to enhance animal resilience to infections.

## Materials and methods

### *Rothia* sequencing and preparation

Total DNA was extracted from the isolated bacteria. Taxonomical classification of this bacterial isolate was previously done after Sanger sequencing of the amplicon obtained through PCR of the 16S gene (primers 8F and 1492R) (Macrogen). Additionally, the extracted DNA was submitted for whole genome sequencing using Illumina MiSeq technology. Raw reads were assembled using Unicycler v0.4.8 (45) and a polish step was performed with Pilon (version 1.23) (46). The draft genome was aligned against several representative genomes from *Rothia* genus and a phylogenetic tree was built with RAxML (47) to confirm the classification. The *R. nasimurium* genome was annotated using RAST (48) tool kit. The resistome was predicted by comparing the assembled contigs with ResFinder vs 4.6.0 (49). For bacteria preparation, the *R. nasimurium* isolated from warthog microbiota and the commercial *Rothia mucilaginosa* (DSMZ; DSM n°20746) were grown overnight on chocolate agar at 37°C with 5% CO2. Then, bacterial suspensions were prepared to achieve the desired multiplicity of infection (MOI) ranging from 1 to 17. Serial dilutions of the final suspension were performed to confirm bacteria concentration. For heat-inactivation of *R. nasimurium*, bacterial colonies were collected, resuspended in PBS, and incubated at 65°C for 1 hour. Before inactivation, an aliquot of the culture was used to quantify the bacteria concentration by serial dilutions. Inactivation was confirmed by absence of overnight growth at 37°C with 5% of CO2 on chocolate agar. For stimulation assays, bacterial suspensions were first centrifuged at 2500 x g 10 min and resuspended in the corresponding media. Heat-inactivated bacteria were used at MOIs ranging from 1 to 50, as indicated in the corresponding figure legends.

### Primary cells isolation

Porcine alveolar macrophages (PAMs) were obtained through lung lavage of healthy animals (from Landrace, Landrace x Duroc and Landrace x Large White pig breeds) using phosphate-buffered saline (PBS) supplemented with 1 µg/ml of gentamicin (Sigma-Aldrich). Cells were maintained in Roswell Park Memorial Institute (PRMI) 1640 medium (Gibco) supplemented with 10% heat-inactivated fetal calf serum (FCS) (Invitrogen), 1% of penicillin-streptomycin/ml (P/S) (Invitrogen), 1% of L-glutamine (Invitrogen), and 0.5% of nystatin (Invitrogen). For the stimulation assays, three to four lots of PAMs were seeded in 96-well flat-bottom plates at 5x10^5^ cells/well and left overnight at 37°C. Next day, cells were incubated with alive or heat-inactivated *Rothia* at the desired concentrations for 24 hours, after which supernatants were collected and stored at -80°C for TNFα detection by ELISA (R&D system). Cell viability was assessed by flow cytometry or using the CellTiter-Glo assay (Promega). Peripheral blood mononuclear cells (PBMCs) obtained from whole blood of healthy animals (Landrace x Duroc) by density-gradient centrifugation using Histopaque 1077 (Sigma-Aldrich). PBMCs were suspended in RPMI 1640 medium supplemented with 10% FCS, 1% P/S, 1% L-glutamine, and 0.05 mM 2-mercaptoethanol.

### RNA-seq library preparation and sequencing

Four lots of PAMs were seeded at 5x10^6^ cells/well in a 6-well flat-bottom plate, and left overnight at 37°C. Cells were stimulated for 6 or 24 hours with lipopolysaccharide (LPS) (InvivoGen; tlrl-eblps) at 10 µg/ml, Pam3CKS (InvivoGen tlrl-pm2s-1) at 10 µg/ml, or heat-inactivated *R. nasimurium* (HI-Ro) at MOI 50. Non-stimulated cells were used as control. Total RNA was isolated using the RNeasy Mini Kit (Qiagen) following the manufacturer’s protocol. To ensure RNA quality, DNase I treatment was performed for 15 min at room temperature. Total RNA was submitted for sequencing to the Centre Nacional d’Anàlisi Genòmica (CNAG), Barcelona, Spain. Total RNA concentration was quantified using Qubit RNA BR Assay kit (ThermoFisher Scientific) and the RNA integrity was estimated by Agilent Bioanalyzer. The RNASeq libraries were prepared with KAPA mRNA HyperPrep Kit (Roche) following the manufacturer’s recommendations starting with 500 ng of total RNA as the input material. The library was quality controlled on an Agilent 2100 Bioanalyzer with the DNA 7500 assay. The libraries were sequenced on NovaSeq 6000 (Illumina) with a read length of 2x51bp, following the manufacturer’s protocol for dual indexing. Image analysis, base calling and quality scoring of the run were processed using the manufacturer’s software Real Time Analysis.

### RNA-seq bioinformatic analysis

Illumina reads were mapped against the genome of *Sus scrofa* (Sscrofa11.1) using STAR software version 2.7.8a (50) with ENCODE parameters. Annotated genes were quantified with RSEM version 1.3.0 (51) with default parameters using the *Sus scrofa* ENSEMBL annotation release 110. Differential expression analysis was performed with the limma v3.42.3 R package (52), using TMM normalization. The voom function (53) was used to transform the count data into log2-counts per million (logCPM), estimate mean-variance relationship and to compute observation-level weights. These voom-transformed counts were used to fit the linear models. Given the paired nature of the data, the individual variation was blocked using the *duplicateCorrelation* function. Contrasts for pairwise comparisons were extracted, as well as contrasts for the interaction effect between treatment and vaccination status. Genes were considered to be differentially expressed (DE) if they had an adjusted p-value <0.05. Functional enrichment analysis was performed using DAVID (http://david.ncifcrf.gov/) (54), considering all DE genes, or the DE genes with an absolute fold change |FC|>1.5, as specified in the corresponding figure legends.

### Inflammasome and innate immune memory assay

For the inflammasome assay, three lots of PAMs were seeded at 5x10^5^ cells/well in a 96-well flat-bottom plate, and left overnight at 37°C. Cells were primed with LPS at 50 ng/ml for 3 hours and then stimulated with HI-Ro at MOI 50 or Imject Alum Adjuvant (ThermoFisher Scientific) at 0.5 mg/ml for 21 hours further. Supernatant was collected and stored at -80°C until IL-1β detection by ELISA (R&D system). For the innate immune memory assay, four to six lots of PAMs were seeded at 1x10^5^ cells/well in a 96-well flat-bottom plate, and left overnight at 37°C. Cells were primed HI-Ro at MOI 1, 5 or 10 for 24 hours and then washed three times with PBS. Fresh media was replaced on day 3. At 6 days post-priming, cells were restimulated with LPS at 10 ng/ml or infected with fluorescence ASFV strains at MOI 0.1. TNFα levels in supernatants were quantified by ELISA (R&D system) at days 1 and 3 post-priming, and at 24 and 48 hours post restimulation or infection.

### Western blot analysis

For immunoblotting, cells were prepared in a 48-well flat-bottom plates at 6.25x10^6^ cells/well. After stimulation as indicated in the previous section, cells were collected with the lysing buffer RIPA buffer (ThermoFisher Scientific) containing protease inhibitor (Thermo Fisher Scientific; 78430) and denatured with 4x LDS loading buffer and 10x reducing agent (Invitrogen; NP0009) at 70°C for 10 min. Subsequently, lysed cells were subjected to NuPAGE 4-12% BisTris gel (Invitrogen) and then transferred onto a Nitrocellulose blotting membrane (Sigma-Aldrich; GE10600003) by electroblotting. Next, membranes were blocked with 1% nonfat dry milk and then stained with anti-IL-1β (R&D system; AF-401-NA) and anti-β-actin (proteintech; 81115-1-RR-100UL). The presence of cytokine was detected by chemiluminescence with a donkey anti-goat-HRP (Santa Cruz Biotechnology; sc-2056) and donkey anti-rabbit-HRP (Cytva; NA934), respecitvely. Imaging was performed with a Fluorochem HD2 chemiluminescent workstation (Alpha Innotech, San Leandro, CA, USA).

### Virus infection of macrophages

The virulent ASFV strain Georgia2007/1 (genotype II) was kindly provided by Dr. Linda Dixon (WOAH reference laboratory, The Pirbright Institute, UK). The attenuated ASFV strain BA71ΔCD2 is a live attenuated vaccine prototype lacking the CD2v gene (*EP402R*), obtained by homologous recombination from the parental virulent ASFV strain BA71 (55). The virulent PRRSV strain Rosalia was isolated from the serum of an infected animal from a farm in Huesca (Spain). All viruses were expanded in PAMs. To evaluate the antiviral activity of HI-Ro, three to four lots of PAMs were defrosted and seeded in a 48-well flat-bottom plate at 6x10^5^ cells/well, and left overnight at 37°C. Next, cells were infected with the Georgia2007/1 ASFV strain at MOI 0.1 or the Rosalia PRRSV strain at MOI 0.05 during two hours in 100 ul/well of RPMI supplemented with 1% L-glutamine. After infection, cells were stimulated with HI-Ro at MOI 50 during 24 hours for Rosalia, or 48 hours for Georgia2007/1. Cells were stained for flow cytometry analysis, and supernatant was collected for TNFα detection by ELISA (R&D system).

### Time-lapse microscopy of viral infection kinetics in macrophage populations

The Georgia2007/1 and BA71ΔCD2 fluorescent viruses used to analyze viral infection kinetics by time-lapse microscopy were obtained by CRISP/R technology by fusing the gene encoding for the mWasabi fluorescent protein (https://www.fpbase.org/protein/mwasabi/) to the C- terminus of the p54 ASFV protein, as previously described (56). To evaluate the capacity of HI-Ro to induce long-term antiviral activity in the innate immune memory assay, at 6 days post-priming, cells were infected with the fluorescence ASFV strains at MOI 0.1 in 50 ul/well of RPMI supplemented with 1% L-glutamine. SYTOX™ Orange Nucleic Acid Stain (Invitrogen) at 50 nM final concentration was added as internal control to detect cell death events following previously published experimental procedures (57, 58). Viral infection kinetics were then quantified over 72 hours by time-lapse microscopy using an IncuCyte^®^ SX5 (Sartorius BioAnalytical Instruments Inc, CA, USA). Specifically, plates were imaged every 2h at 20x (4 fields of view per well) using the device’s “AI Scan” module in three channels (phase, GFP, Orange) using default acquisition parameters (GFP acquisition time 300 ms, Orange acquisition time 400 ms). Cell detection was performed using the “AI Cell Health” module (Segmentation Sensitivity = 0.7) without filtering for cell size. Classification of detected cells across the dimensions “uninfected/infected” was finally performed using the “Cell-by-Cell Classification” module: infected cells were identified based on a Green Mean Intensity threshold of 1 GCU (set empirically to have ~99% of cells in uninfected controls shown as negative).

### Phagocytosis assay using pHrodo Cell labeling kit

*Glaesserella parasuis* strain SW114 bacteria (59) were labeled with the pH sensitive dye pHrodo™ according to the manufacturer’s instructions (4766; Sartorius). Briefly, cells were grown overnight on chocolate agar at 37°C with 5% CO2 reaching 10^8^ colony-forming unit/ml. After washing, pHrodo was added at 1000 µg/ml and incubated 1h at 37°C, and cells were washed and resuspended in the complete RPMI used for PAMs culture. For the phagocytic assay, PAMs were cultured at 5x10^4^ cells/well in a 96 well flat-bottom plate, and left overnight at 37°C. Cells were stimulated for 2 or 24 hours with HI-Ro at MOI 10 or 50, LPS at 10 or 0.1 µg/ml, or left non-stimulated as control. After stimulation, pHrodo-labelled bacteria were added to the plate at MOI 50. pHrodo positive cells were then quantified over 24 hours by time-lapse microscopy using an IncuCyte^®^ SX5 (Sartorius BioAnalytical Instruments Inc, CA, USA). Specifically, plates were imaged every 30min at 20x (4 fields of view per well) using the device’s “AI Scan” module in two channels (Phase and Orange) using default acquisition parameters. Cell detection was performed using the “AI Cell Health” module (Segmentation Sensitivity = 0.7) without filtering for cell size. Classification of detected cells across the dimensions “non-phagocytosis/phagocytosis” was finally performed using the “Cell-by-Cell Classification” module: phagocytic cells were identified based on an Orange Mean Intensity threshold of 0.1.

### Flow cytometry and sorting

Flow cytometry was performed as previously described (60). Briefly, PAMs and PBMCs were seeded at 6x10^5^ or 20x10^6^ cells/well, respectively. Cells were stained for viability with LIVE/DEAD Fixable Violet Dead Cell Stain Kit, LIVE/DEAD Fixable Red Dead Cell Stain Kit, Fixable LIVE/DEAD Fixable Near-IR Stain Kit, or DAPI (4′,6-diamidino-2-phenylindole), according to the manufacturer’s instructions (ThermoFisher Scientific). Blockage of Fc receptors was performed with PBS containing 5% of porcine serum (Gibco) for 15 min on ice prior to antibody staining. For PAMs extracellular staining, cells were incubated with anti-SLA II DR (Bio-Rad Laboratories; MCA2314A647) and/or anti-CD80 (Invitrogen; 62-0801-82). For sorting of macrophages and CD21b+ B cells, four lots of PBMCs were stained with anti-CD14 (Bio-Rad; MCA1218GA) and anti-CD21b (MA5-28322; Invitrogen). The purity of sorted cells was >95% for both populations. Purified monocytes and CD21b+ B cells were resuspended at 1x10^5^ or 3x10^6^ cells/ml respectively, and stimulated overnight with HI-Ro at MOI 50. Cells were then stained with anti-CD14, anti-CD21b, anti-SLA II DR and anti-CD80 extracellular antibodies. For intracellular staining of virus-infected PAMs, cells were fixed and permeabilized with the BD Cytofix/Cytoperm Kit (BD Biosciences) according to the manufacturer’s protocol, and incubated during 30 min on ice in Perm/Wash buffer with anti-p72 antibody (Eurofins Ingenasa; M.11.PPA.I1BC11) for ASFV, or the anti-N protein antibody (clone 1CH5) (Ingenasa; M.11.PRS.I1CH5) for PRRSV. Then, cells were incubated with the secondary antibodies anti-mouse IgG1 (eBioscience; 25-4015-82) and anti-mouse IgG2b (Jackson ImmunoResearch; 115-165-207), respectively. In all cases, samples were acquired in a BD FACSAria IIu flow cytometer (BD Biosciences) and data was analyzed using FlowJo v10.8.1 software (Tree Star Inc).

### Statistical analyses

Graphs were created and analyzed using Prism version 8.0.2 software (GraphPad), R 4.3.3 and RStudio version 2022.07.2 software. Statistical tests used are indicated in the corresponding figure legends. Statistical significance was set at p < 0.05 (ns p > 0.05; *p ≤ 0.05; **p ≤ 0.01; ***p ≤ 0.001; ****p ≤ 0.0001).

## Results

### *Rothia nasimurium* stimulates cytokine production in various porcine immune cells

**Figure 1:**
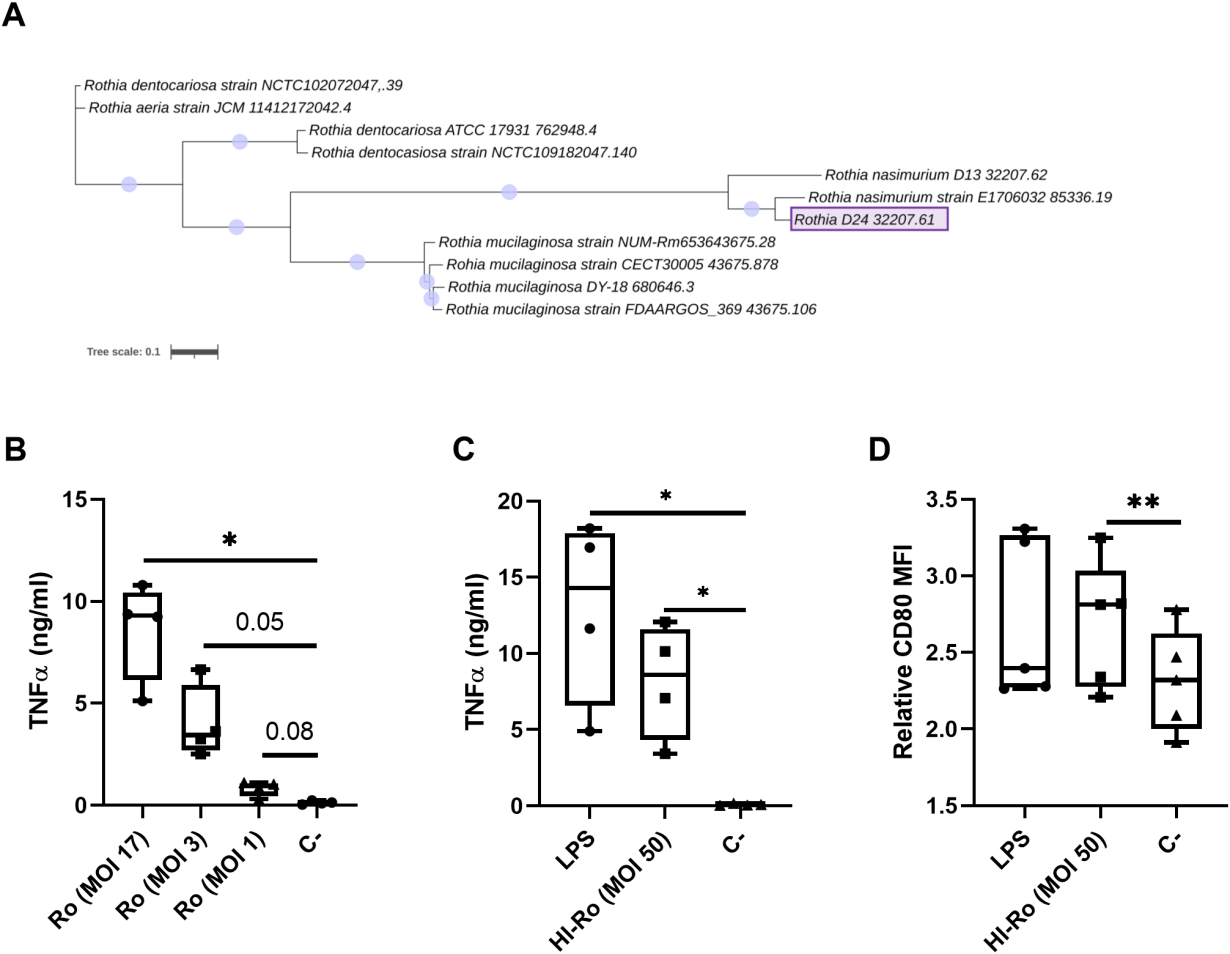
Treatment of porcine alveolar macrophages with alive (Ro) or heat-inactivated *Rothia nasimurium* (HI-Ro) induces the production of the pro-inflammatory cytokine TNFα. **(A)** Phylogenetic tree of available full genomes of *Rothia* spp. The *Rothia* strain isolated from warthog microbiota is depicted as *Rothia* D24 32207.61. The scale bar corresponds to 0.1 change per nucleotide. **(B–D)** PAMs were stimulated for 24 hours with alive Ro **(B)** or HI-Ro **(C, D)** at the indicated MOIs. Levels of TNFα in cell supernatants **(B, C)** were measured by ELISA, and the mean fluorescence intensity (MFI) of CD80 in SLA-II+ macrophages was analyzed by flow cytometry **(D)**. LPS-stimulated (10 µg/ml) or non-stimulated cells were used as positive and negative controls, respectively. Significant differences were determined using a one-way ANOVA with p-values of **≤ 0.01 and *≤ 0.05.

**Figure 2:**
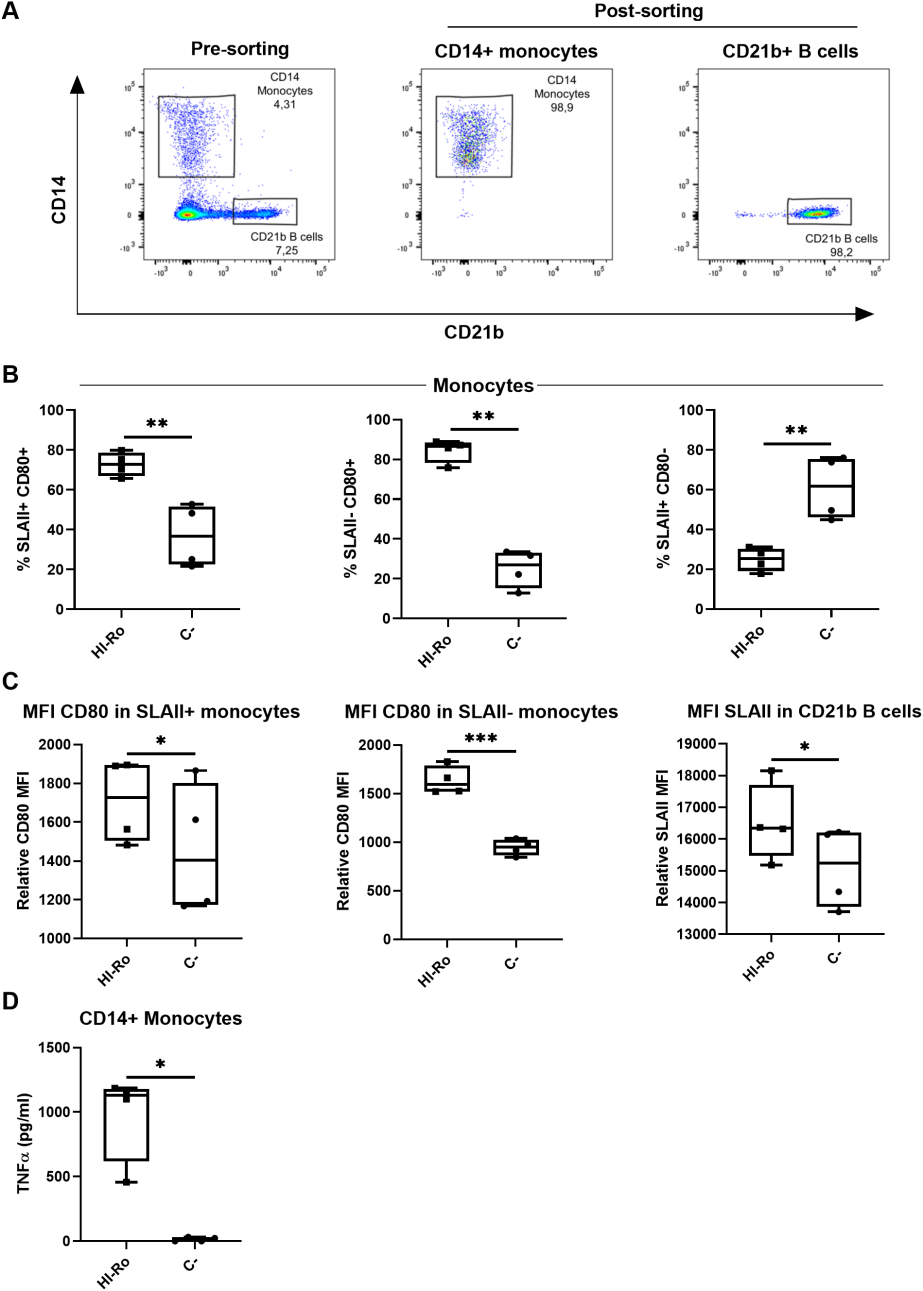
Heat-inactivated *Rothia nasimurium* (HI-Ro) activates porcine monocytes and CD21b+ B cells. **(A)** Representative flow cytometry plots showing the purified monocyte and CD21b+ B cell populations. **(B–D)** Purified monocytes or CD21b+ B cells were stimulated for 18 hours with HI-Ro at MOI 50. Percentages of SLAII+CD80+, SLAII-CD80+ and SLAII+CD80+ monocytes **(B)**, and the mean fluorescence intensity (MFI) of CD80 in monocytes or SLAII in CD21b+ B cells **(C)**, were analyzed by flow cytometry. Levels of TNFα in supernatants from HI-Ro stimulated monocytes **(D)** were measured by ELISA. Significant differences were determined using a T-test with p-values of ***≤ 0.001 **≤ 0.01 and *≤ 0.05.

In a previous study we isolated a bacterium from warthog microbiota which was identified as *Rothia* spp. by 16S rRNA sequencing, and showed potential as immunostimulant by inducing IFN-γ secretion in GALT cell suspensions (44). Here we aimed to further characterize the immunostimulatory capability of this bacteria strain in porcine alveolar macrophages (PAMs). First, we performed full genome sequencing by Illumina MiSeq technology to further characterize the bacteria species. Based on the sequencing results, the bacterium was classified as *Rothia nasimurium*, as shown in the corresponding phylogenetic tree (**Figure 1A**). Next, we validated the capability of this strain of *R. nasimurium* to induce cytokine production. The bacterium induced TNFα production (**Figure 1B**) without affecting cell viability (**Supplementary Figure 1A**). Moreover, heat inactivation of the bacterium did not affect either its stimulatory profile or viability, as demonstrated by the secretion of TNFα and the upregulation of CD80 at 24 hours post-stimulation, similar to LPS-stimulated cells (**Figures 1C, D** and **Supplementary Figure 1B**). To investigate if heat-inactivated *R. nasimurium* (HI-Ro) is also capable to activate other antigen presenting cells, we next stimulated purified blood monocytes and CD21b+ B cells (**Figure 2A**). HI-Ro stimulation resulted in an increase of the percentage of CD80+ monocytes and its mean fluorescent intensity (**Figures 2B, C**), as well as in TNFα production (**Figure 2D**). CD21b+ B cells also were activated upon stimulation as demonstrated by the increase of SLAII mean fluorescence intensity (**Figure 2C**). Importantly, the viability of both cell subsets was not affected by HI-Ro stimulation (**Supplementary Figures 1C, D**). Overall, these results demonstrate the capability of both live and HI-Ro to activate PAMs, and thus its potential as immunostimulant.

### Heat-inactivated *Rothia nasimurium* fully activates the inflammasome

**Figure 3:**
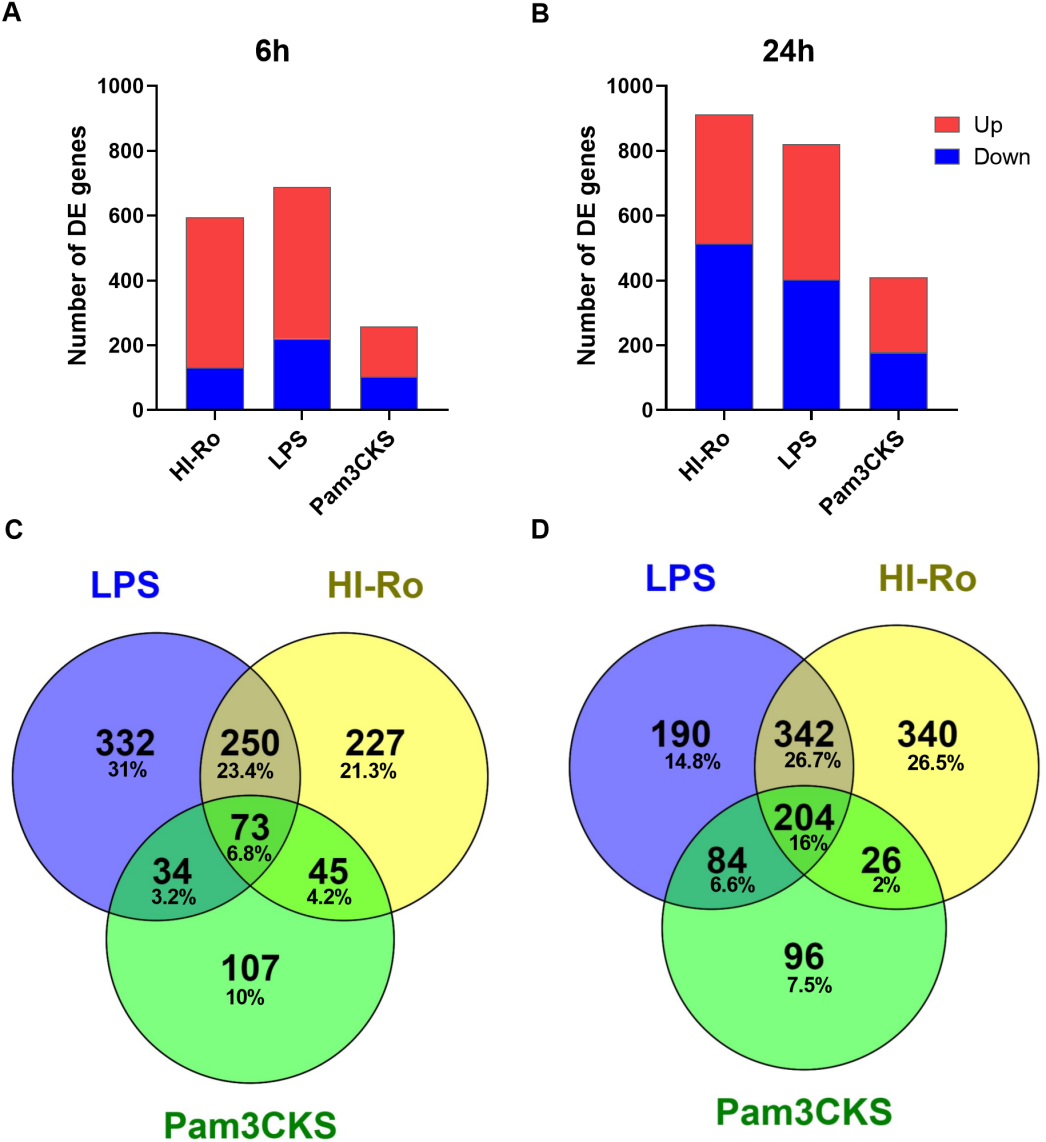
Heat-inactivated *Rothia nasimurium* (HI-Ro) induces robust transcriptomic changes in porcine alveolar macrophages. **(A, B)** Number of RNA-seq-derived DE genes with an absolute fold change |FC| > 1.5 up- or down-regulated at 6 **(A)** or 24 **(B)** hours post-stimulation with HI-Ro (MOI 50), LPS (10 µg/ml), or Pam3CKS (10 µg/ml). Non-stimulated cells were used as reference. **(C, D)** Venn diagrams showing the number and percentage of shared or unique DE genes (|FC| > 1.5) for each treatment at 6 **(C)** or 24 **(D)** hours.

**Figure 4:**
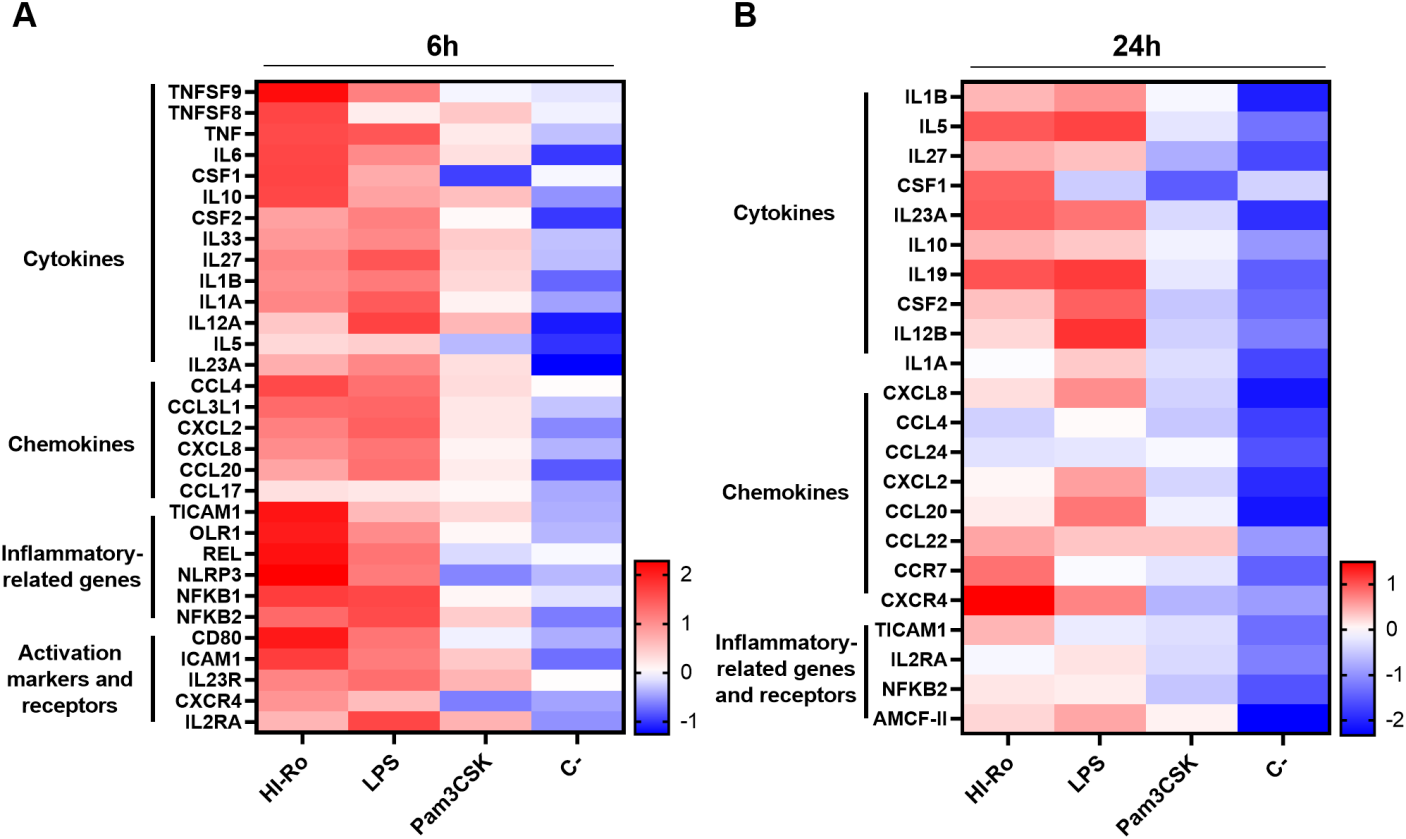
Porcine alveolar macrophages treated with heat-inactivated *Rothia nasimurium* (HI-Ro) reveals a robust activation of the innate system. Heatmaps depicting the z-score from normalized RNA-seq-derived log2CPM values of representative DE genes in the categories indicated with an absolute fold change |FC| > 1.5 at 6- **(A)** and 24-hours **(B)** post-stimulation with HI-Ro (MOI 50), LPS (10 µg/ml), or Pam3CKS (10 µg/ml).

**Figure 5:**
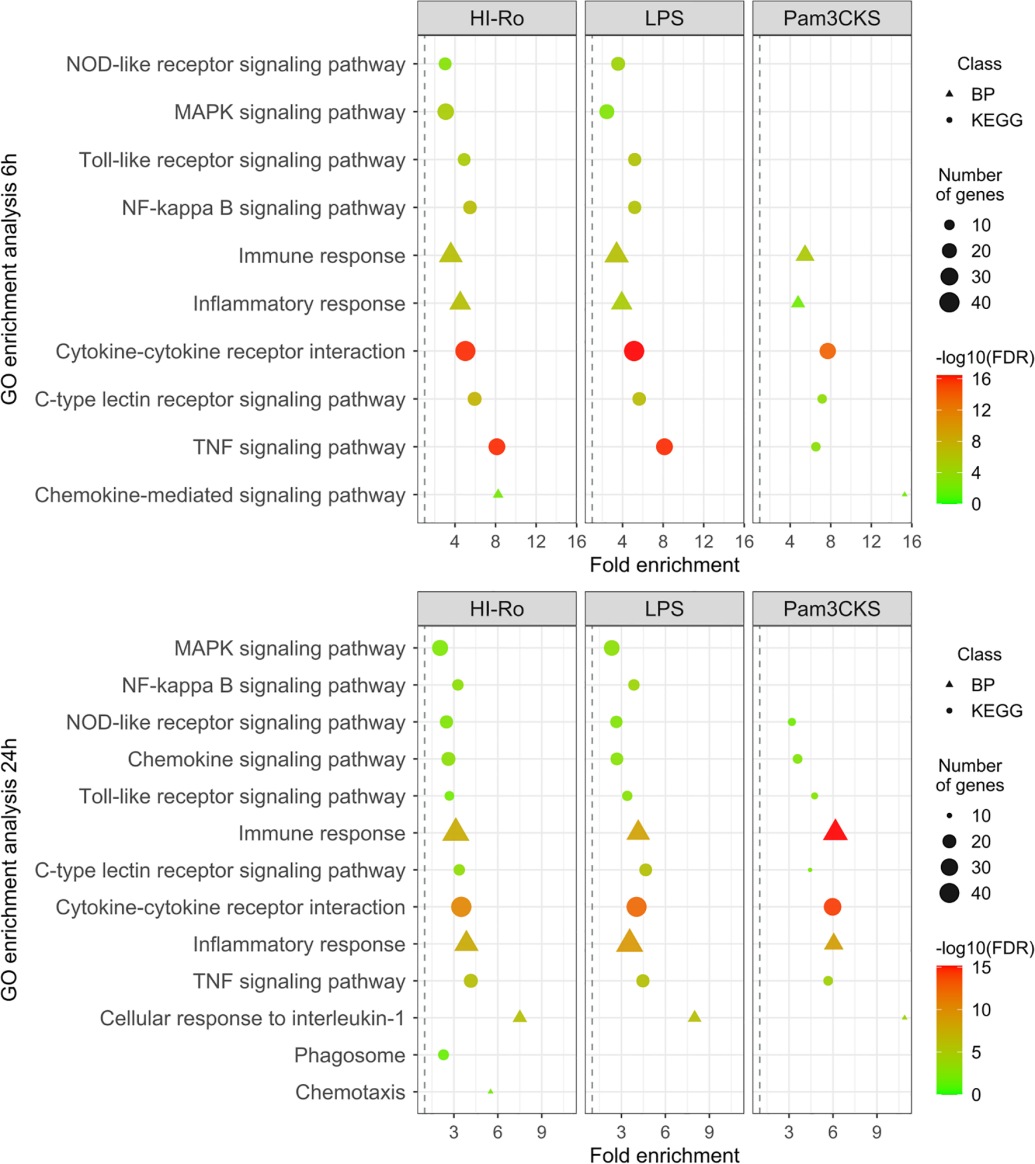
Gene ontology (GO) enrichment analysis showing the immunostimulatory capacity of heat-inactivated *Rothia nasimurium* (HI-Ro) in porcine alveolar macrophages. List of representative GO terms enriched in DE genes with an absolute fold change |FC| > 1.5 from PAM stimulated during 6 or 24 hours with HI-Ro (MOI 50), LPS (10 µg/ml), or Pam3CKS (10 µg/ml). Non-stimulated cells were used as reference. The shape indicated the GO database (the Kyoto Encyclopedia of Genes and Genomes (KEGG) or biological process (BP)). The dot size represents the number of DE genes associated with the GO term. The dot color indicates the negative log10 value of the false discovery rate (FDR).

**Figure 6:**
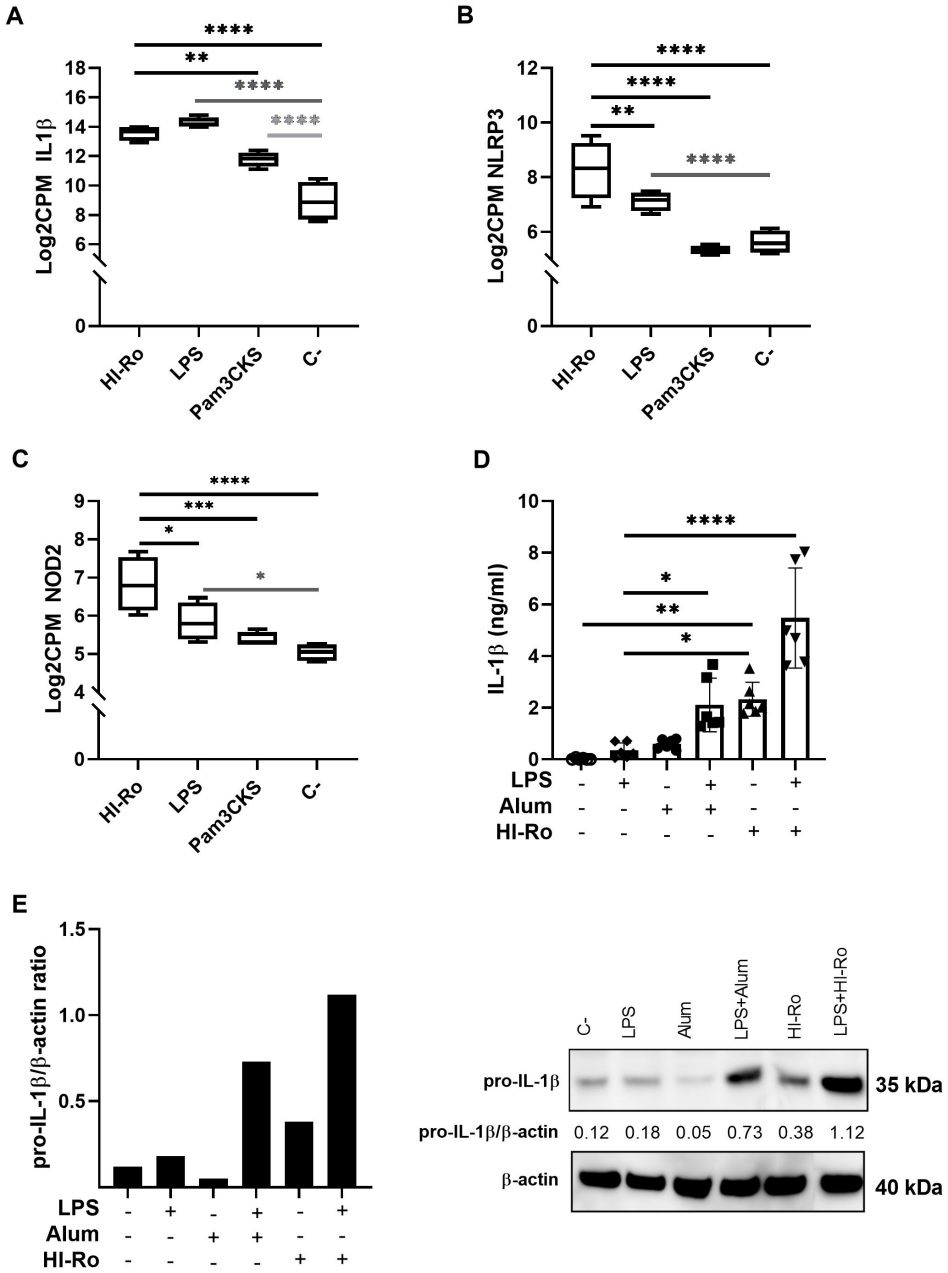
Heat-inactivated *Rothia nasimurium* (HI-Ro) promotes the production of IL-1β upon inflammasome activation in porcine alveolar macrophages. **(A–C)** RNA-seq-derived expression levels of *IL1β* **(A)**, *NLRP3* **(B)**, and *NOD2* **(C)** in PAMs stimulated with HI-Ro (MOI 50), LPS (10 µg/ml) or Pam3CKS (10 µg/ml). Non-stimulated cells were used as negative control (C-). **(D, E)** Levels of mature IL-1β in cell supernatants **(D)** and pro-IL-1β in lysate cells **(E)** were quantified from PAMs primed with LPS (50 ng/ml) for 3h, and next stimulated with HI-Ro (MOI 50) or Alum (0.5 mg/ml) for 21 hours. **(D)** Mature IL-1β levels in supernatants were measured by ELISA. All conditions were run in technical duplicates. Significant differences were determined using a one-way ANOVA. **(E)** AWestern blot assay was performed to quantify pro-IL-1β and β-actin production from cell lysates. Representative significant differences are indicated with p-values of ****≤ 0.0001, ***≤0.001, **≤ 0.01 and *≤ 0.05.

To further investigate the stimulatory capacity of HI-Ro, we next characterized the transcriptomic changes induced in treated PAMs. Cells obtained from four different animals were stimulated with HI-Ro for 6 or 24 hours, and their transcriptome profile obtained by RNA-seq was compared to PAM stimulated with the TLR4 ligand LPS or the TLR1/2 ligand Pam3CKS. Differentially expressed (DE) genes (adjusted p-values<0.05) were obtained taking non-stimulated samples as a reference, and filtered with an absolute fold change |FC|>1.5 for further analyses. HI-Ro and LPS treated cells showed a larger number of DE genes at both time points compared to Pam3CKS (**Figures 3A, B** and **Supplementary Figure 2**). Moreover, HI-Ro and LPS also showed a higher percentage of shared genes (**Figures 3C, D**), thus indicating a similar induction of signaling pathways with both treatments. The stimulatory capacity of HI-Ro was validated by the robust upregulation of genes encoding cytokines, chemokines, activation markers, receptors and other genes related to inflammation (**Figure 4**). To further understand these transcriptomic changes, we next performed a gene ontology enrichment analysis. Again, HI-Ro- and LPS-treated cells were enriched with genes belonging to similar biological processes and pathways at both time points. These included several terms related to innate immunity, such as NF-kβ, MAPK, TLR and C-type lectin signaling pathways (**Figure 5**), thus validating the immunostimulatory properties of HI-Ro. Interestingly, among the different enriched terms, we also identified the inflammasome-associated NOD-like receptor signaling pathway, which has been related to the efficacy of aluminum adjuvants (61). Importantly, although the three tested treatments induced a significant upregulation of *IL1β* expression (**Figure 6A**), cells stimulated with HI-Ro showed a higher expression of the *NLRP3* and the *NOD2* genes compared to LPS (**Figures 6B, C**). Since these two genes are involved in the activation of caspase-1 and the consequent production of IL-1β and interleukin-18 (IL-1β) (62–64), we next investigated the capability of HI-Ro to promote the production of pro- and mature IL-1β by activating the inflammasome, as previously demonstrated by aluminum adjuvants (65). As previously reported (61), priming of PAMs with a low dose of LPS was not enough to induce production of mature IL-1β in supernatants, and Alum-stimulated macrophages only secreted mature IL-1β when previously primed with LPS (**Figure 6D**). Importantly, LPS-primed cells produced even higher amounts of IL-1β when stimulated with HI-Ro. Interestingly, macrophages stimulated with the bacteria alone produced higher amounts of both pro- and mature IL-1β in contrast to Alum alone (**Figures 6D, E**), indicating that HI-Ro acts both as priming and activating signals. Altogether, these results demonstrate the capacity of *R. nasimurium* to induce a robust innate immune response in porcine macrophages, including the activation of the inflammasome acting both as a priming and activation signal.

### Antimicrobial properties of HI-Ro-treated macrophages

**Figure 7:**
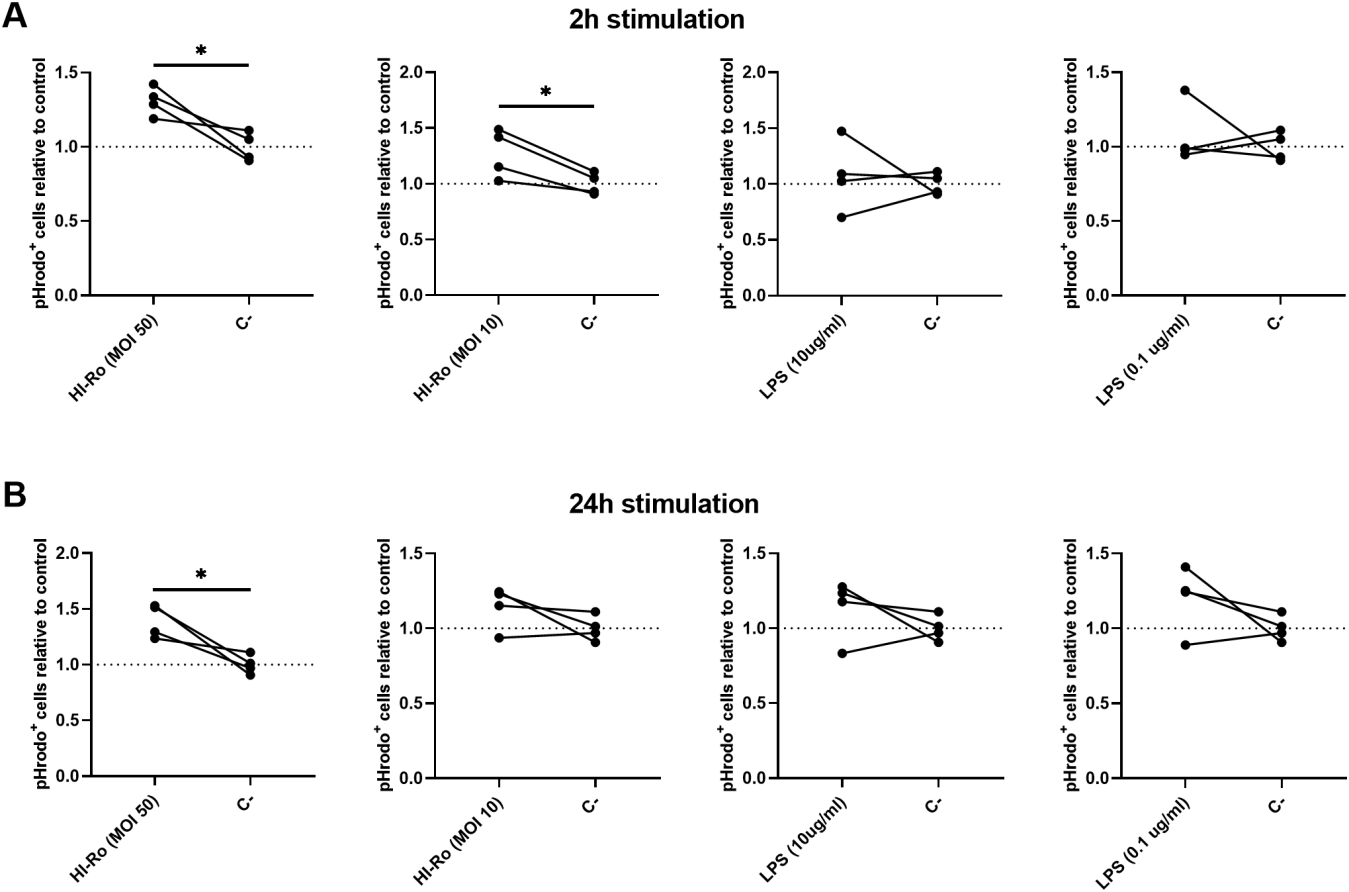
Alveolar macrophages treated with heat-inactivated *Rothia nasimurium* (HI-Ro) exhibit an enhanced phagocytic activity towards *Glaesserella parasuis*. **(A, B)** PAMs were stimulated for 2 **(A)** or 24 **(B)** hours with HI-Ro at MOIs 10 or 50, and LPS at 0.1 or 10 µg/ml. Non-stimulated cells were used as control (C-). Statistical differences in the percentages of phagocytic cells (pHrodo^+^) relative to non-stimulated control cells were assessed for each condition at 24 hours post-exposure with *G*. *parasuis*. Significant differences were determined using t-test with p-values of *≤ 0.05.

**Figure 8:**
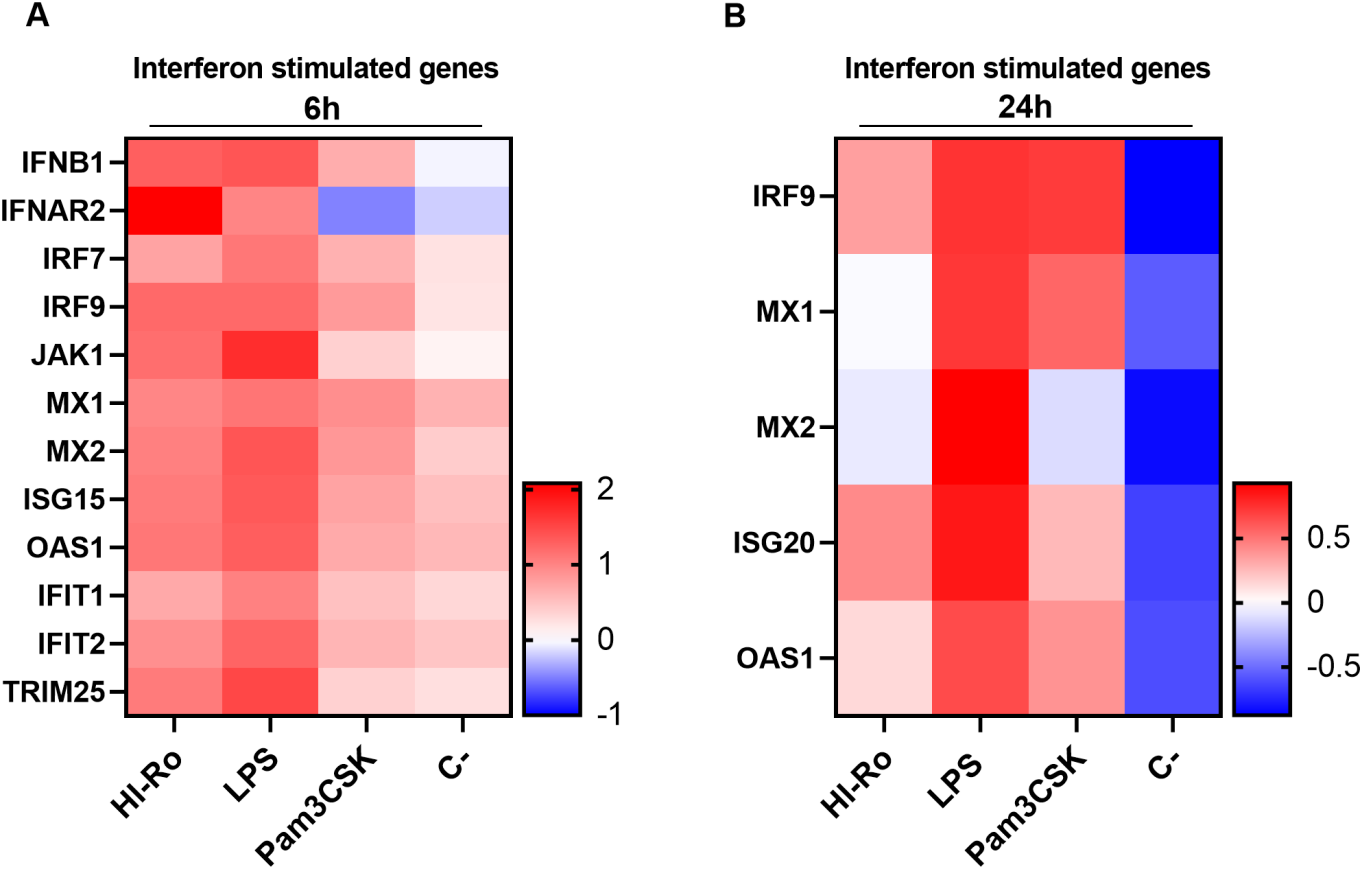
Expression levels of interferon stimulated genes (ISG) in porcine alveolar macrophages. **(A, B)** Heatmaps showing the z-score from normalized RNA-seq-derived log2 CPM values of differentially expressed (adjusted p-values<0.05) ISG at 6- **(A)** and 24-hours **(B)** post-stimulation with heat-inactivated *R. nasimurium* (HI-Ro; MOI 50), LPS (10 µg/ml) or Pam3CKS (10 µg/ml).

**Figure 9:**
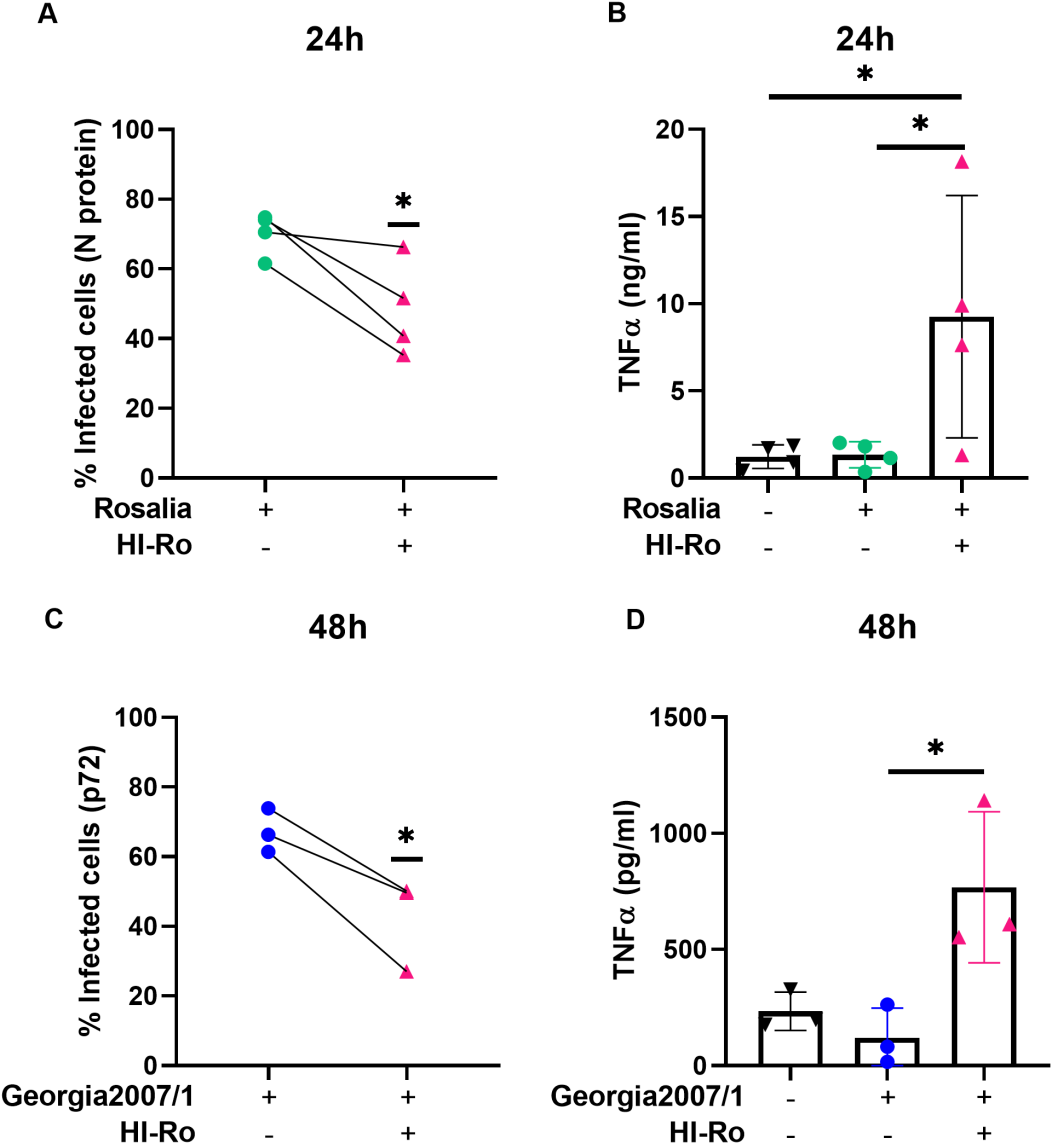
Treatment of PRRSV- or ASFV-infected porcine alveolar macrophages with heat-inactivated *Rothia nasimurium* (HI-Ro) reduces viral replication. Porcine alveolar macrophages were infected with the PRRSV strain Rosalia **(A, B)** or the ASFV strain Georgia2007/1 **(C, D)**. Two hours post-infection cells were treated with HI-Ro (MOI 50). At the indicated time points, percentages of infected cells were measured by flow cytometry **(A, C)**, and levels of TNFα in supernatants were quantified by ELISA **(B, D)**. Non-infected cells were used as negative control. Significant differences were determined using t-test **(A, C)** or one-way ANOVA **(B, D)** with p-values of *≤ 0.05.

We next investigated if the activation of alveolar macrophages by HI-Ro-treatment prints them with an antimicrobial functional state. One of the main physiological functions of alveolar macrophages is the phagocytosis of inhaled particles and/or pathogens to maintain pulmonary homeostasis (66). Thus, we first analyzed the capability of HI-Ro-treated PAMs to phagocyte the bacteria *Glaesserella parasuis*, causing agent of Glässer disease in pigs. PAMs were treated with HI-Ro or LPS, and exposed to fluorescently-labelled *G. parasuis* at 2- or 24-hours post-treatment. The percentage of fluorescent macrophages, indicating active phagocytosis of the bacterium, was quantified in real time by Incucyte (**Supplementary Figure 3**). Importantly, while at initial time points there were not differences in the phagocytic capability of treated and untreated cells, at late time points HI-Ro treatment resulted in a significant increase in the percentage of phagocytic macrophages (**Figure 7** and **Supplementary Figure 3**). Besides the enhanced phagocytic capability of HI-Ro-treated macrophages, the innate immune responses triggered by the bacterium in PAMs likely results in the acquisition of an antiviral state, thus increasing their resilience to infection. Indeed, RNAseq-derived data showed the upregulation of type I interferon (IFN-I) and interferon stimulated genes (ISG) in HI-Ro-treated macrophages (**Figure 8**), indicating an antiviral state. To further evaluate this issue, we next tested the capacity of HI-Ro to damper the replication capacity of two major porcine viral pathogens infecting macrophages: the porcine reproductive and respiratory syndrome virus (PRRSV) and the African swine fever virus (ASFV). PAMs were infected *in vitro* with the highly virulent virus strains PRRSV Rosalia (MOI 0.05), or ASFV Georgia2007/01 (MOI 0.1). At two hours post-infection, cells were treated with HI-Ro or left untreated, and infected cells were quantified by flow cytometry. Demonstrating the antiviral properties acquired by HI-Ro-treated macrophages, the percentage of PRRSV- or ASFV-infected cells were significantly lower in treated cells than untreated cells at 24 or 48 hours post-infection, respectively (**Figures 9A, C**). These differences were not linked to changes in the viability of treated and untreated infected cells (**Supplementary Figure 4**). Moreover, this reduction of virus infectivity was concomitant to a change of the immunological context of infected cell cultures. Indeed, the amount of TNFα in supernatants from infected cell cultures was significantly higher in HI-Ro-treated samples, indicating an improvement of the functional immune status of infected cells (**Figures 9B, D**).

**Figure 10:**
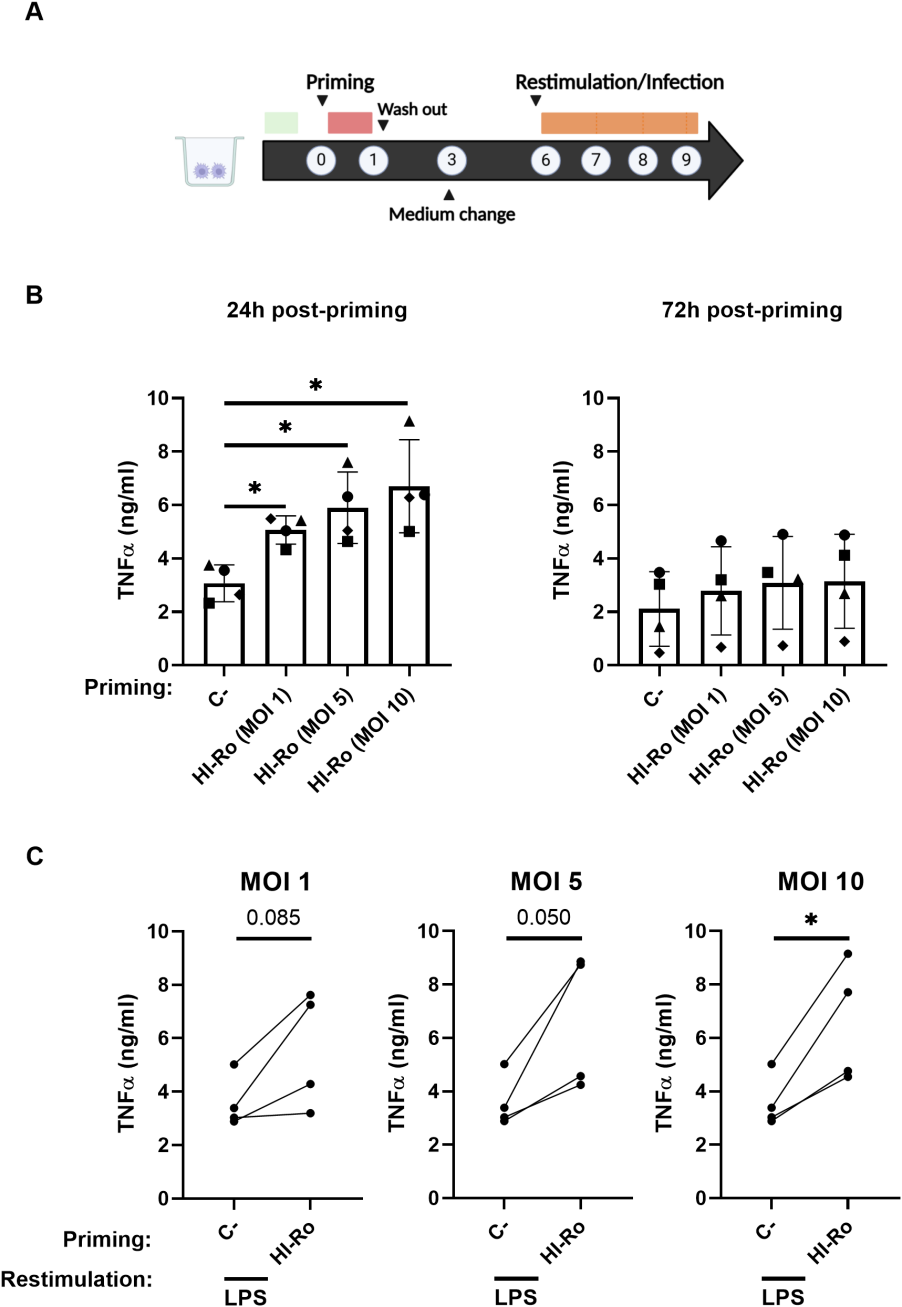
Heat-inactivated *Rothia nasimurium* (HI-Ro) induces an enhanced long-term nonspecific response. **(A)** Schematic representation of the *in vitro* procedure performed. **(B, C)** Porcine alveolar macrophages were primed during 24 hours with HI-Ro at the indicated MOIs. Non-stimulated cells were used as negative control (C-). Levels of TNFα in cell supernatants measured by ELISA at 24 and 72 hours post-priming **(B)**, and at 24 hours post-restimulation with LPS (10 ng/mL) **(C)**. Significant differences were determined using one-way ANOVA **(B)** or t-test **(C)** with p-values of *≤ 0.05. Created with BioRender.com.

**Figure 11:**
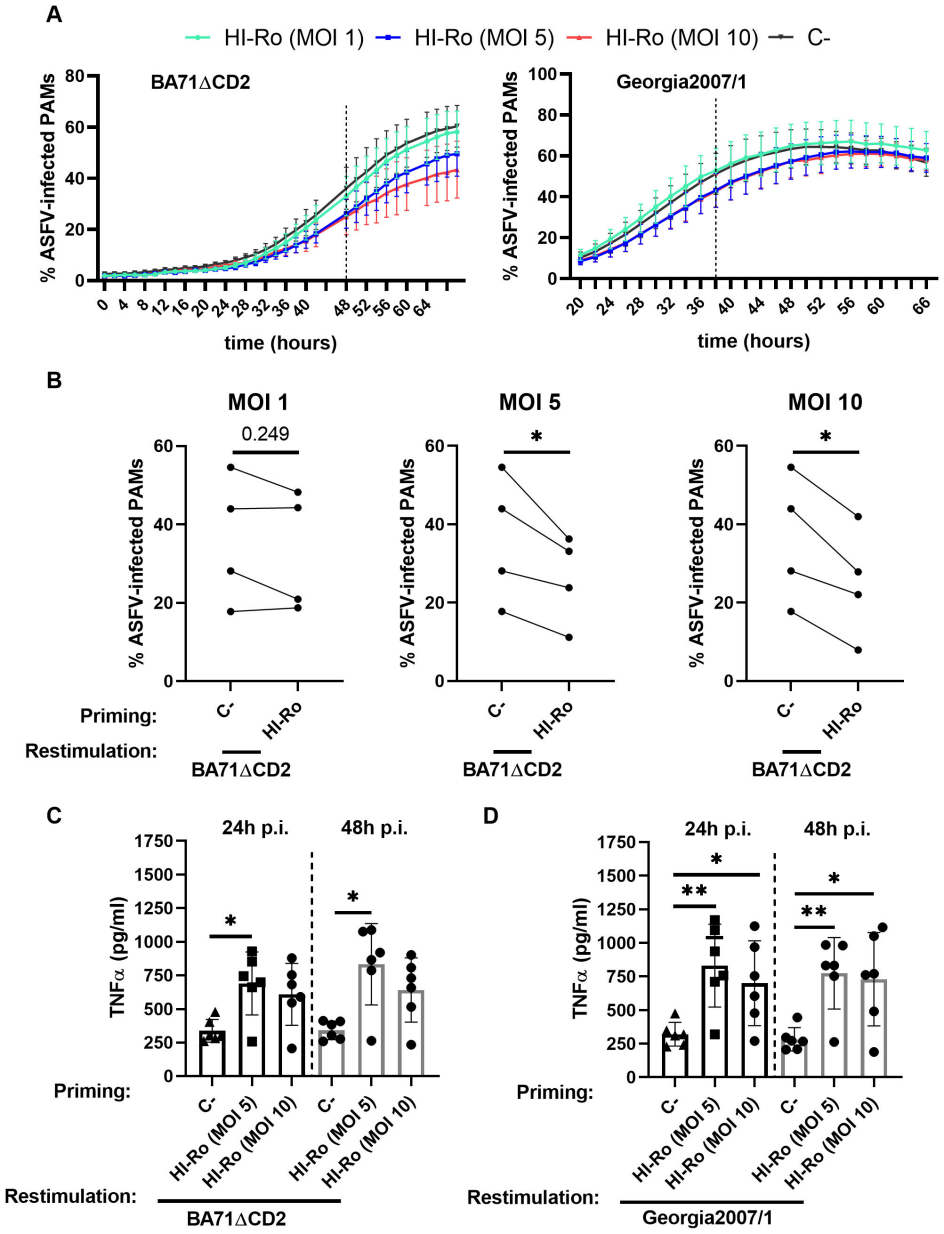
Priming with heat-inactivated *Rothia nasimurium* (HI-Ro) reduces the susceptibility of macrophages to BA71ΔCD2 ASFV infection. Porcine alveolar macrophages (PAMs) were primed during 24 hours with HI-Ro at the indicated MOIs. Non-stimulated cells were used as negative control (C-). Six days after priming, cells were infected with fluorescent-labelled attenuated (BA71ΔCD2; **A**–**C**) or virulent (Georgia2007/1; **A, D**) ASFV strains. **(A)** Percentages of ASFV-infected cells analyzed every 2 hours by Incucyte. **(B)** Statistical differences between primed and unprimed cells were assessed for each MOI at 48 hours post-infection with BA71ΔCD2. **(C, D)** Levels of TNFα in supernatants at 24- and 48-hours post-infection (p.i.) with BA71ΔCD2 **(C)** or Georgia2007/1 **(D)** quantified by ELISA. Significant differences were determined using t-test **(B)** or one-way ANOVA **(C, D)** with p-values of **≤ 0.01, *≤ 0.05.

We next evaluated whether HI-Ro treatment of macrophages induces long-lasting functional changes characteristic of innate immune memory, resulting in enhanced antiviral responses reducing their susceptibility to future infections. To address this issue, PAMs were treated with HI-Ro, and 6 days later were restimulated with LPS following the schedule illustrated in **Figure 10A**. As the induction of innate immune memory is achieved at low doses (67), we tested the effect of priming PAMs with three different low HI-Ro doses (MOI 1, 5 and 10). Cells stimulated with these HI-Ro doses induced a significant cytokine production at 24 hours post-stimulation, returning to a resting state at 72 hours post-stimulation (**Figure 10B**). Importantly, cells primed with HI-Ro showed an innate memory phenotype, producing higher amounts of TNFα at 24 hours post-restimulation with LPS than untreated cells (**Figure 10C**). To evaluate if this higher responsiveness has consequences in terms of susceptibility to infection, we repeated the experiment priming with HI-Ro, and infecting treated cells with the highly lethal Georgia07 or the attenuated BA71ΔCD2 ASFV strains (55) at day 6 post-priming. To assess real-time virus expansion kinetics, we obtained by CRISPR/Cas9 technology two recombinant viruses encoding for a fluorescent protein (mWasabi) fused to the ASFV p54 structural protein, and the number of infected cells was monitored every few hours using time-lapse microscopy (68). The expansion kinetics of the attenuated BA71ΔCD2 ASFV strain was significantly reduced in cells previously primed with HI-Ro at MOIs 5 and 10, showing lower fractions of virus-infected cells over time (**Figures 11A, B**). To note, this effect was observed in all macrophage lots at MOI 10, while a higher variability was observed at MOI 5 (**Supplementary Figure 5**). These differences were not observed in cells infected with the highly virulent Georgia07 ASFV strain (**Figure 11A** and **Supplementary Figures 6, 7**). However, a tendency to decrease the number of infected cells upon treatment with HI-Ro was observed at 38 hours post-infection. Importantly, virus infection with both strains also resulted in an enhancement of TNFα production at 24- and 48-hours post-infection by HI-Ro-primed macrophages (**Figures 11C, D**), demonstrating the capacity of infected cells to overcome the virus-induced innate immune suppression characteristic of ASFV. Overall, these results demonstrate the capacity of HI-Ro to induce long-lasting functional modification to alveolar macrophages, reducing their susceptibility to ASFV infection while enhancing their antiviral responsiveness.

## Discussion

The use of immunostimulants with the capacity to enhance animal resilience to diseases has become a promising approach to reduce the incidence of infections in livestock. However, the veterinary pharmaceutical industry demands cost-effective products with good safety profiles to reach the market. Here we have identified a novel inactivated bacteria strain that fulfils these criteria, and demonstrated its capability to modulate porcine innate immune cells *in vitro*, imprinting them with enhanced antimicrobial functions. The results obtained clearly demonstrate that the heat-inactivated *Rothia nasimurium* strain obtained from warthog fecal microbiota (44, 69) has potential as a vaccine adjuvant, and reduces macrophage susceptibility to at least two of the major current porcine viruses’ threats. Moreover, we demonstrated *in vitro* the potential of heat-inactivated *R. nasimurium* to induce innate immune memory in porcine alveolar macrophages, a phenotype that might provide long-term heterologous protection against infections. While these *in vitro* studies demonstrate the immunostimulatory capability of *R. nasimurium* on porcine immune cells, further *in vivo* studies will be required to evaluate its potential to enhance animal resilience to disease.

Our results are in line with previous studies showing the immunostimulatory capacity of the strain *R. dentocariosa*, which induces NF-kB activation and TNFα production by a human monocyte cell line (42). However, this is in contrast with another study demonstrating an anti-inflammatory activity of *R. mucilaginosa* in LPS-stimulated epithelial cells (43), as well as with recent results obtained with *R. nasimurium* isolated from domestic pig’s nasal microbiota (41). These variable immunomodulatory properties indicate a context-dependent outcome upon *Rothia* cell stimulation. Indeed, here we show that neither *R. nasimurium* nor *R. mucilaginosa* have anti-inflammatory properties in LPS-stimulated porcine alveolar macrophages (**Supplementary Figure 8**), demonstrating differential bacteria-host interactions depending on the cells involved. This complexity in the outcome derived from a potential *Rothia*-based treatment is further increased when considering its safety profile. While most *Rothia* strains are part of the healthy microbiota, some strains can cause opportunistic infections (40). For instance, *R. dentocariosa* is frequently associated with dental caries and periodontal disease (70), and eventually it can also cause invasive disease (71, 72). To avoid these eventual issues, our studies used a safe version of the bacteria based on heat-inactivation with higher probabilities to reach the market. The inactivation of the bacteria did not damper its immunostimulatory capabilities, indicating that the underlying molecular compounds are not affected in heat-killed *Rothia*. Interestingly, antigenic glycolipids have been isolated from different *Rothia* strains (73, 74). Thus, the thermoresistance of bacterial glycolipids (74), together with their significant stimulatory properties on macrophages (75, 76), rank them as the one of the bacterial components responsible for the immunostimulatory capacity of *Rothia*. However, further studies will be necessary to characterize the molecular components responsible of the broad activation of macrophages observed upon *Rothia* stimulation, probably involving the triggering of various PRRs.

Inflammasome activation has a critical role in innate immunity, initiating a broad inflammatory response through the release of the cytokines IL-1β and IL-18, and the activation of apoptotic and pyroptotic cell death. These effects have been associated with the enhancement of vaccine responses mediated by Alum particulates adjuvants (61, 65). However, the relative contribution of this inflammasome activation in Alum-mediated induction of adaptive immune responses is controversial, depending on the vaccination schedules tested (77, 78). Nevertheless, these findings resulted in an increasing interest in the discovery of novel vaccine adjuvants based on inflammasome- and TLR targeting compounds (6, 79). Indeed, it has been shown that TLR-activation by monophosphoryl lipid A, a component of the AS04 adjuvant which also includes aluminum, is critical to enhance the Th1 response, thus improving the adjuvant effect compared to aluminum alone (80). The broad innate immune signaling pathways triggered by heat-inactivated *R. nasimurium* position it as a good adjuvant candidate. Indeed, besides the TLR-mediated activation shown in the transcriptomic analysis, we demonstrate a superior capacity than Alum to induce both pro- and mature IL-1β production through inflammasome activation. Further studies *in vivo* will be required to test this hypothesis with appropriate vaccine candidates, as well as extended *in vitro* analyses evaluating the stimulatory capability of *R. nasimurium* in dendritic cells, a critical innate immune cell subset for vaccine efficacy. Given the importance of adjuvant discovery to enhance animal and human vaccines, the identification of cost-effective and safe immunostimulatory compounds such as *R. nasimurium* is becoming a relevant research line in vaccinology (9, 73).

In addition to IFN-I response, inflammasome activation is also critical to eliminate pathogens during infections (81), playing a particular role in antiviral responses. Indeed, several viruses have developed immune scape strategies to evade IFN-I and inflammasome activation in their benefit (82, 83). Thus, the activation of these innate immune pathways might underline the antiviral activity shown by inactivated *R. nasimurium* in porcine alveolar macrophages infected with PRRSV and ASFV. Indeed, both viruses are sensitive to IFN-I treatment (84–86), trigger similar immune responses upon infection of macrophages (87), and have evolved strategies to evade innate immunity (88–92). Therefore, the activation of innate immunity in infected cells by immunostimulants might counteract these suppressive mechanisms resulting in a diminished viral replication capacity. However, the reduced viral expansion could also be a consequence of a nonpermissive state of uninfected bystander alveolar macrophages, due to a direct *R. nasimurium*-stimulation or an indirect cytokine-mediated modulation of their phenotype. Indeed, both PRRSV and ASFV have a reduced replication capacity in proinflammatory M1 macrophages (93, 94), thus supporting this hypothesis. Further analyses are required to decipher the mechanisms underlying the antiviral properties shown by inactivated *R. nasimurium*, as well as its feasibility as an antiviral drug *in vivo*. The use of an antiviral compound to treat ASFV-infected pigs seems unlikely due to the highly lethal nature of the disease, which results in the slaughtering of animals in affected farms. This limitation is supported by the little literature studying the effect of anti-ASFV drugs *in vivo* (95, 96), although the appearance of low virulent ASFV strains in Asia is increasing. In the case of PRRSV, due to the important limitations of the current vaccines to control disease spreading, there is an increasing interest in the identification of effective anti-viral drugs, some of them targeting the activation of innate immunity (97–100). Such drugs might help in curtailing the transmission of PRRSV on affected farms, thus having an important impact on disease spreading. Therefore, inactivated *R. nasimurium* or similar compounds might represent a cost-effective approach to minimize the incidence or PRRSV infection in affected farms, reinforcing vaccination programs in the disease management plans. In addition, we have shown that *R. nasimurium* also enhances the phagocytic capability of alveolar macrophages, a critical function which helps in the maintenance of lung homeostasis and the clearance of pathogens (66). Importantly, our results demonstrate that treatment with *R. nasimurium* results in a higher percentage of phagocytic macrophages against *G.parasuis*, an important pathogen for the swine industry (101). To note, the real time analysis performed using the Incucyte methodology allowed us to identify long-term effects on the cell phagocytic capability, as previously shown by others (102), highlighting the importance to analyze the kinetics rather than single time points. However, other *G.parasuis* strains with high resistance to phagocytosis, or other pathogenic bacteria of interest must be tested to further evaluate the beneficial effect of *R. nasimurium* on the alveolar macrophages phagocytic capacity.

The modulation of innate immune responses through the induction of innate immune memory is an increasingly interesting approach to enhance livestock resilience to diseases (103, 104). The long-term and heterologous nature of this response, based on epigenetic modifications, makes it optimal to reduce infection incidences in a cost-effective manner (17). However, in contrast with research performed in humans and mouse models, very few studies have addressed this issue in animal health. Thus, it is important to identify biological compounds inducing innate immune memory in farm animals and investigate their potential contribution in controlling the incidence of diseases affecting livestock. Our results demonstrate that *in vitro* treatment of porcine alveolar macrophages with heat-inactivated *R. nasimurium* induces long-term functional modifications resulting in the enhancement of the innate immune responses against heterologous stimulus. The transcriptomic data suggests that this response is NOD2-dependent, as demonstrated with other models (105). Importantly, we show that treated cells are less susceptible to infection with an attenuated ASFV strain, reducing its replication capacity while enhancing the capacity of macrophages to produce inflammatory cytokines during infection. The *in vitro* system used to evaluate innate immune memory mimics the previously described using human and mouse cells, testing biological components which have also demonstrated its efficacy *in vivo* (106, 107). Similarly, trained immunity induced *in vitro* in bovine monocytes by the Bacillus Calmette-Guérin vaccine was validated *in vivo* in treated calves (108). Thus, although the results obtained here are limited to *in vitro* studies, they suggest the beneficial potential of treating pigs with *R. nasimurium*. However, the use of immunostimulants has to be carefully evaluated before its broad implementation in the field, evaluating potential negative effects that cannot be investigated *in vitro* assays which lack the complexity of real physiological conditions. Indeed, an enhanced innate immune response in treated animals can result in a detrimental persisting and excessive inflammation in particular contexts (109). Importantly, there are already examples of similar bacterial compounds enhancing resilience to disease in livestock animals through the induction of trained immunity. For instance, the administration of the commercial immunostimulant Amplimune (based on mycobacterial cell wall fractions) has a positive impact on health and production in cattle (31–35, 39), and heat-inactivated *Mycobacterium bovis* protects against Salmonellosis in pigs (30). Further research is necessary to investigate if *R. nasimurium* has similar properties than these approved immunostimulants *in vivo*. To note, the potential effect of these bacterial-based compounds in PRRS and ASF disease severities has not been evaluated yet. Thus, the *in vivo* validation of the antiviral properties obtained *in vitro* would represent a differential feature of *Rothia* compared with the existing alternatives. Finally, the animal genetic background also might distinctly affect the innate immune responses induced by *Rothia* stimulation (110, 111), and thus it will also need to be evaluated. Indeed, our *in vitro* assays have been done with porcine alveolar macrophages obtained from different pig lines. This is particularly important when analyzing the *Rothia*-induced anti-PRSSV effects, since it is well described that host genetics significantly influence the susceptibility to PRRSV infection (112, 113).

The *in vivo* testing of *R. nasimurium* will need to address several important issues, such as the inoculation route and the analysis of central versus peripheral innate immune memory (114). Targeting of alveolar macrophages through intranasal inoculation might confer superior protection against relevant respiratory diseases, such as influenza and PRRSV infections. To note, the intranasal administration of vaccines in livestock has gained attention due to the efficiency inducing mucosal immunity (60, 108, 115, 116). Importantly, generation of innate immune memory upon intranasal vaccination with inactivated bacteria has been demonstrated (20, 21), and its induction in mouse alveolar macrophages have been proven both *in vitro* and *in vivo* (106, 117–120). Therefore, the intranasal treatment of pigs with heat-inactivated *R. nasimurium* might be effective. Further *in vivo* studies will be required to evaluate cross-protective responses against relevant pathogens, as well as to analyze potential adverse effects (121). Finally, besides the long-term antiviral activity on treated cells, it will be also interesting to analyze this response results in an enhancement of vaccine efficacy *in vivo*. Indeed, it has been suggested that innate immune memory can improve vaccine-induced immune responses (122). Thus, in the case of the prototype vaccine BA71ΔCD2 tested here, the higher production of inflammatory cytokines, together with a reduction of the replication levels of the prototype vaccine BA71ΔCD2, might result in a positive balance increasing both vaccine efficacy and its safety.

In conclusion, this work characterizes the immunomodulatory capability of a novel bacteria-based immunostimulant with potential to be used as a vaccine adjuvant, as well as a therapeutic or preventive treatment against porcine diseases. Our results demonstrate that treatment with heat-inactivated *R. nasimurium* imprint in alveolar macrophages robust and prolonged modifications enhancing their innate immune responses to heterologous threats. The two viruses used here, PRRSV and ASFV, are two of the major viral infections affecting the porcine industry, causing enormous economic losses worldwide, and thus are significant viral models. Further studies will be required to evaluate the effectiveness of *R. nasimurium* treatment *in vivo*. The use of such immunostimulants in livestock represent a promising strategy to enhance animals’ resilience to diseases, thus improving their sanitary status by reducing the incidence of zoonotic and non-zoonotic infections, which has significantly increased due to globalization and reduction of antibiotics usage.

## Data Availability

The datasets presented in this study can be found in online repositories. The names of the repository/repositories and accession number(s) can be found below: https://www.ncbi.nlm.nih.gov/geo/, GSE288520, https://www.ncbi.nlm.nih.gov/genbank/, JBLKPV000000000.
