# Supplementary material for: Inactivated Rothia nasimurium promotes a persistent antiviral immune status in porcine alveolar macrophages
Source: Front Immunol. 2025 Jun 3;16:1584092. doi: 10.3389/fimmu.2025.1584092 (PMC12170322; doi:10.3389/fimmu.2025.1584092)
Supplement: Supplementary file 1 [file DataSheet1.docx]

Supplementary Material

# Supplementary Figures

**
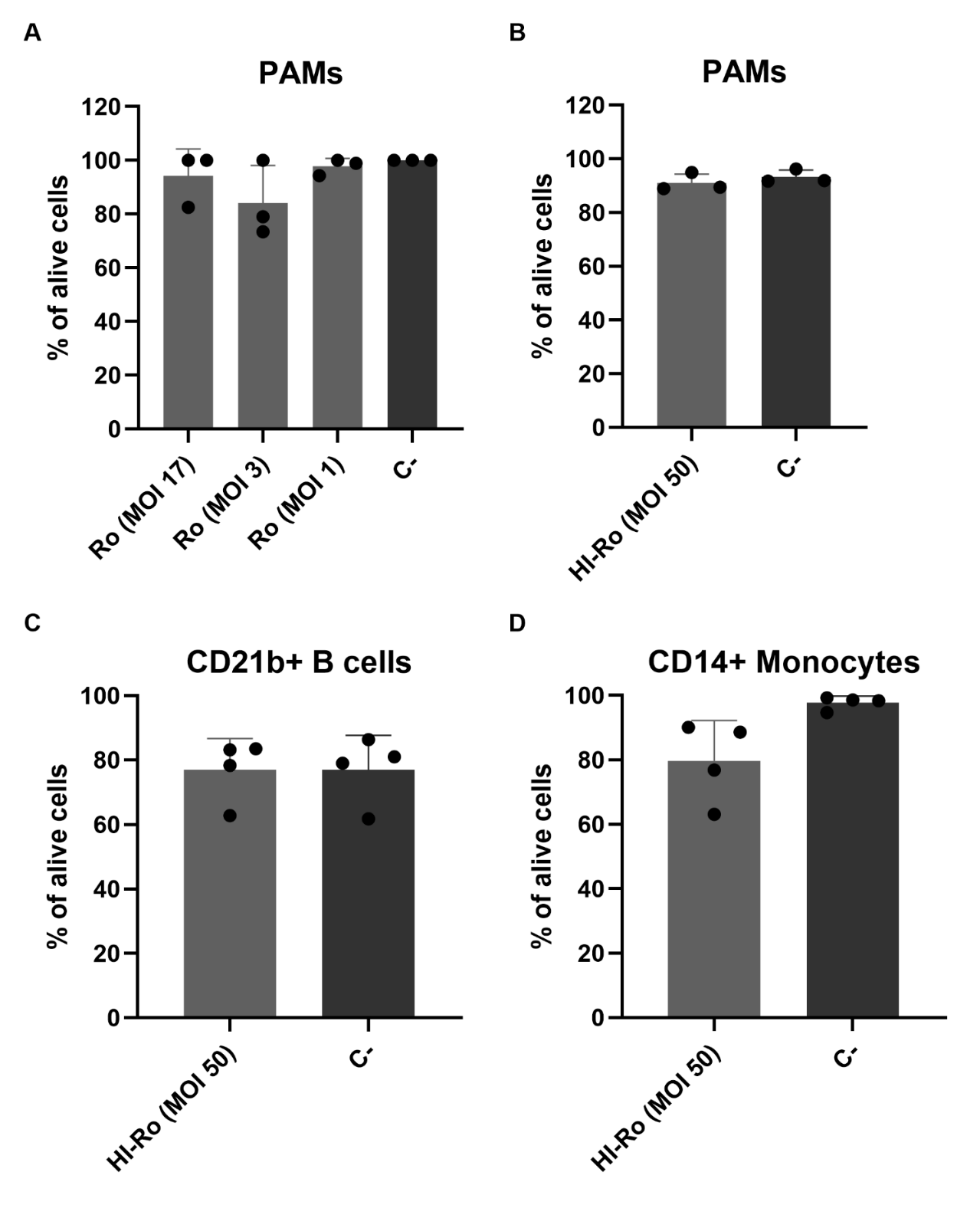
**

**Figure S1. *Rothia nasimurium* does not affect cell viability of different porcine immune cells.** (**A-D**) PAMs, sorted CD14+ monocytes, and CD21b+ B-cells were stimulated during 24 hours with alive *R. nasimurium* (Ro; **A**) or heat-inactivated *Rothia* (HI-Ro; **B-D**) at the indicated MOIs. Non-stimulated cells were used as negative control. Percentages of alive cells were measured using CellTiter-Glo (**A**) or flow cytometry (**B-D**).

**
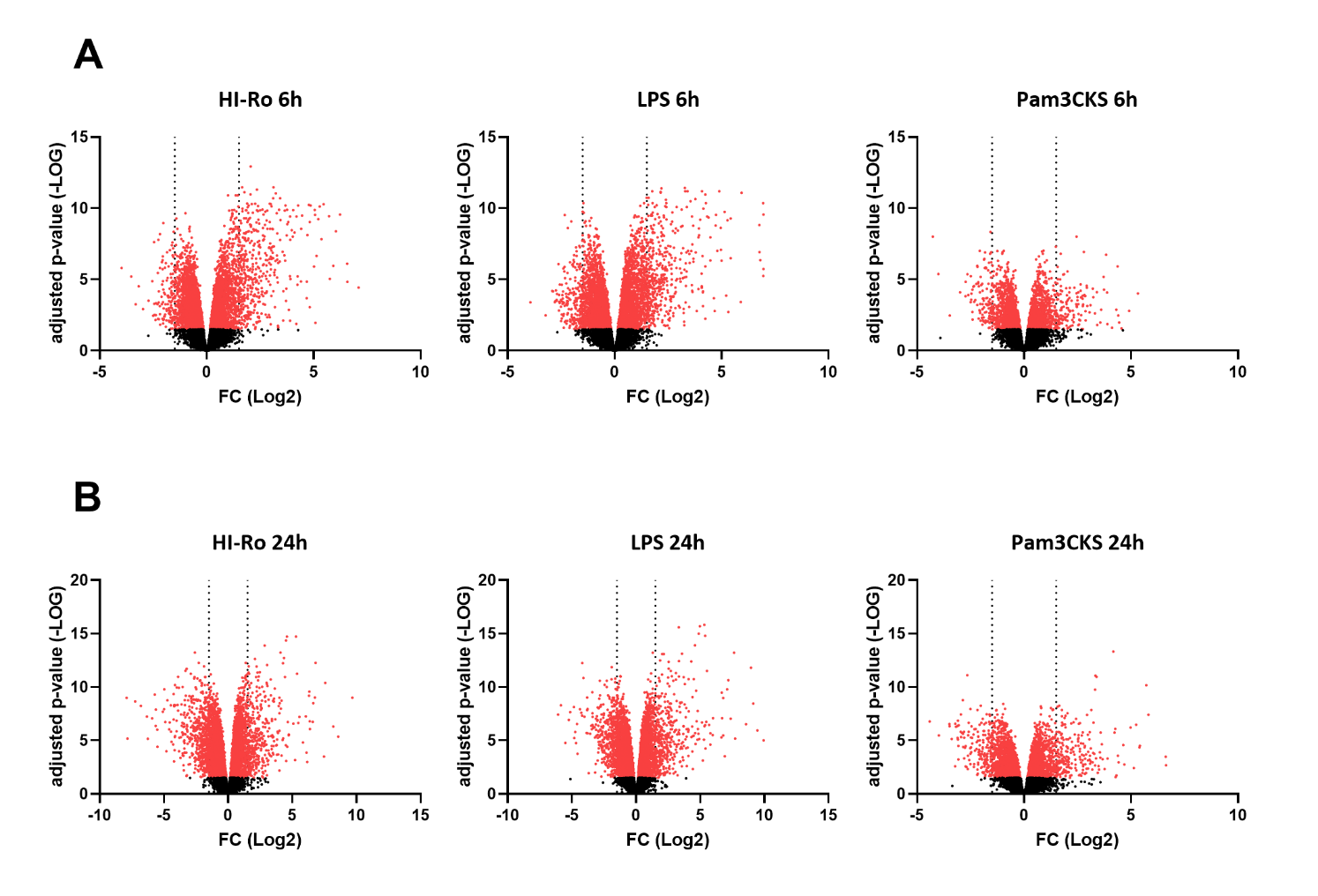
Figure S2. Differentially expressed (DE) genes.** Volcano plots showing fold changes and adjusted p-values for DE genes at 6- (**A**) and 24-hours (**B**) post-stimulation with heat-inactivated *R. nasimurium* (HI-Ro; MOI 50), LPS (10 µg/ml) or Pam3CKS (10 µg/ml). Dashed line in the x axis indicates the absolute fold change |FC| > 1.5.

**
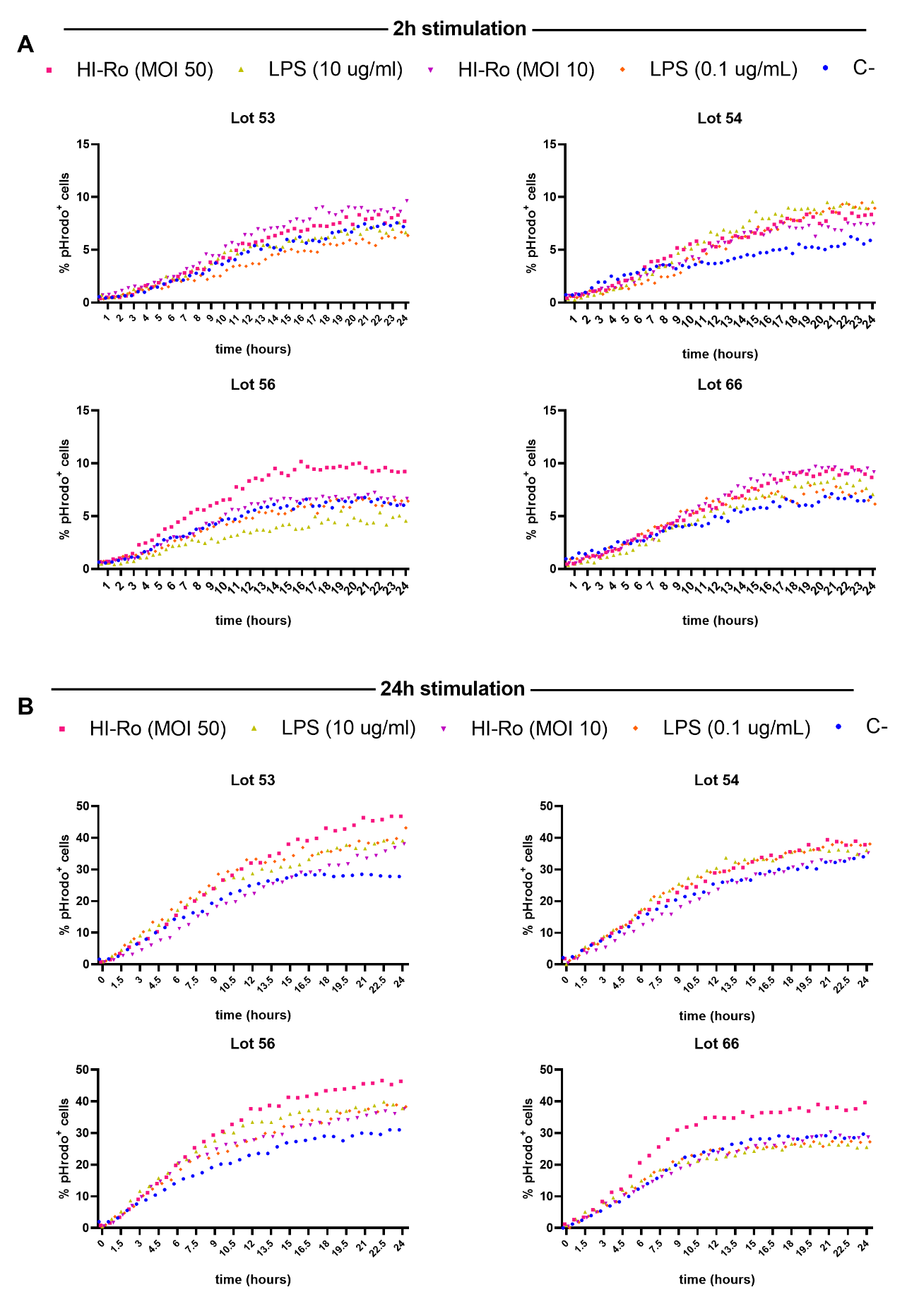
**

**Figure S3. Percentages of phagocytic alveolar macrophages analyzed every 30 minutes by Incucyte.** Data coming from 3 different PAM lots (53, 54, 56, and 66). Cells were stimulated for 2 (**A**) or 24 (**B**) hours with HI-Ro at MOIs 10 or 50, or LPS at 0.1 or 10 µg/ml. Non-stimulated cells were used as control (C-).

**
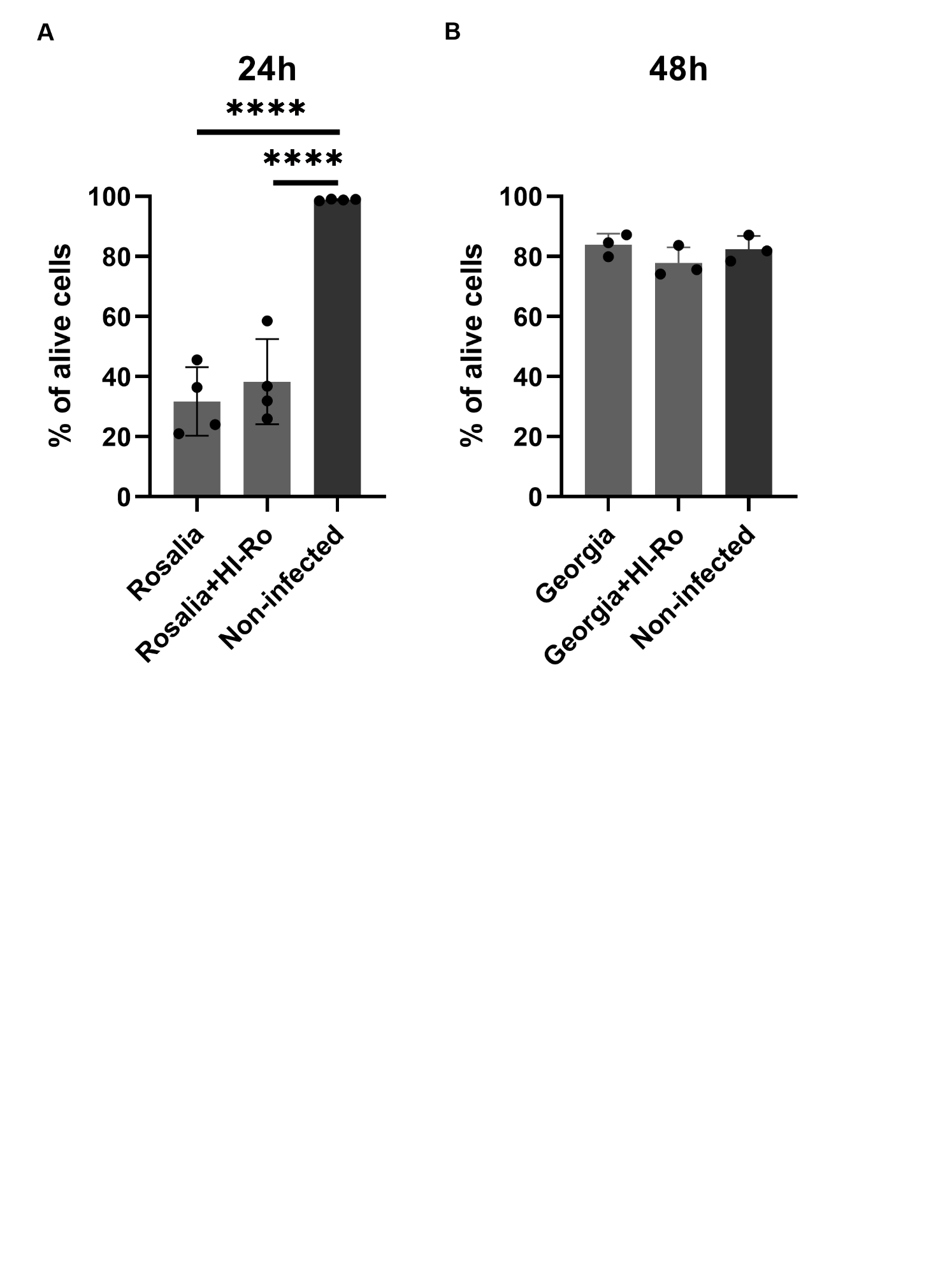
 Figure S4. Heat-inactivated *Rothia nasimurium* (HI-Ro) does not affect cell viability after PRRSV or ASFV infection.** PAMs were infected with the PRRSV strain Rosalia (**A**), or the ASFV strain Georgia2007/1 (**B**). Two hours post-infection, cells were treated with HI-Ro (MOI 50). At the indicated time points, percentages of alive cells were measured by flow cytometry. Non-infected cells were used as negative control. Significant differences were determined using a one-way ANOVA with p-values of ****≤ 0.0001.


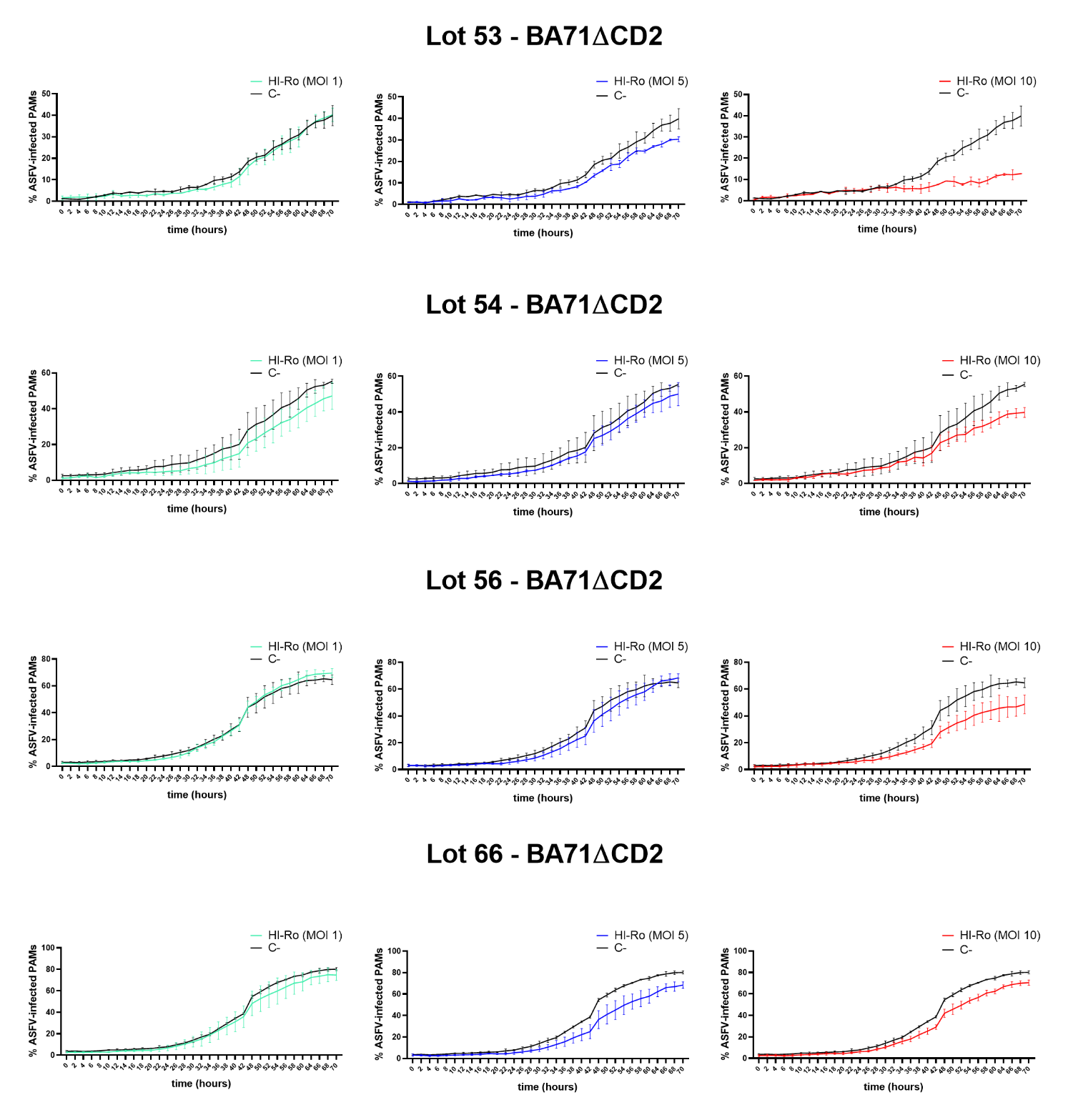


**Figure S5. Percentages of BA71ΔCD2 fluorescent-labelled infected cells analyzed every 2 hours by Incucyte for every individual porcine alveolar macrophage lot.** Data coming from 3 experimental replicates grouped by PAM lot (53, 54, 56, and 66) and treatment concentration (HI-RO MOI 1, 5, and 10). Non-infected cells were used as negative control. Values represent the mean ± SD for each condition.

**
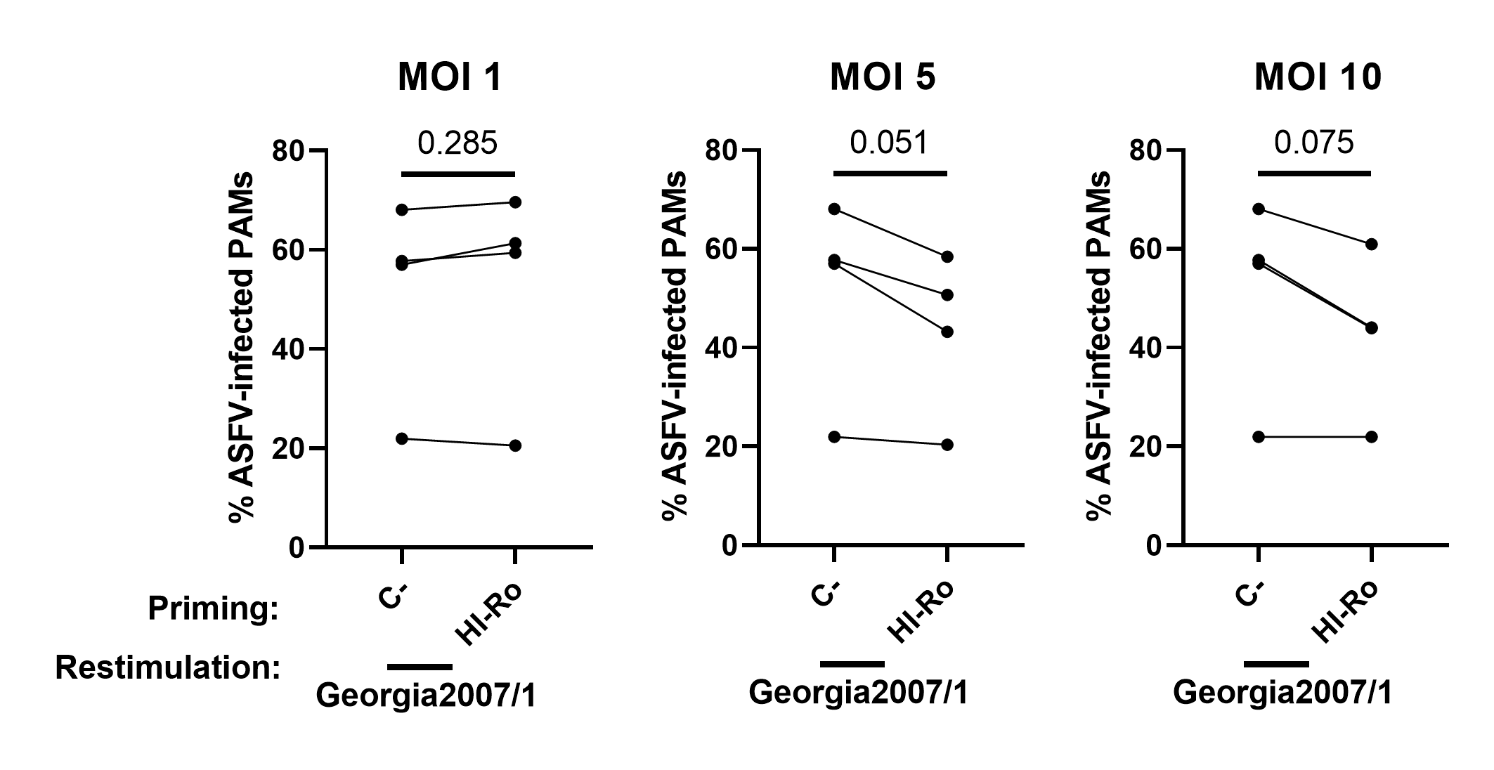
 Figure S6. Priming with heat-inactivated *Rothia nasimurium* (HI-Ro) does not significantly reduce Georgia2007/1 infection.** PAMs were primed during 24 hours with HI-Ro at indicated MOIs. Non-stimulated cells were used as negative control (C-). Six days after priming, cells were infected with a fluorescent-labelled virulent strain (Georgia2007/1). Statistical differences between primed and unprimed cells were assessed for each MOI at 38 hours post-infection. Significant differences were determined using t-test with p-values of ns> 0.05.

**
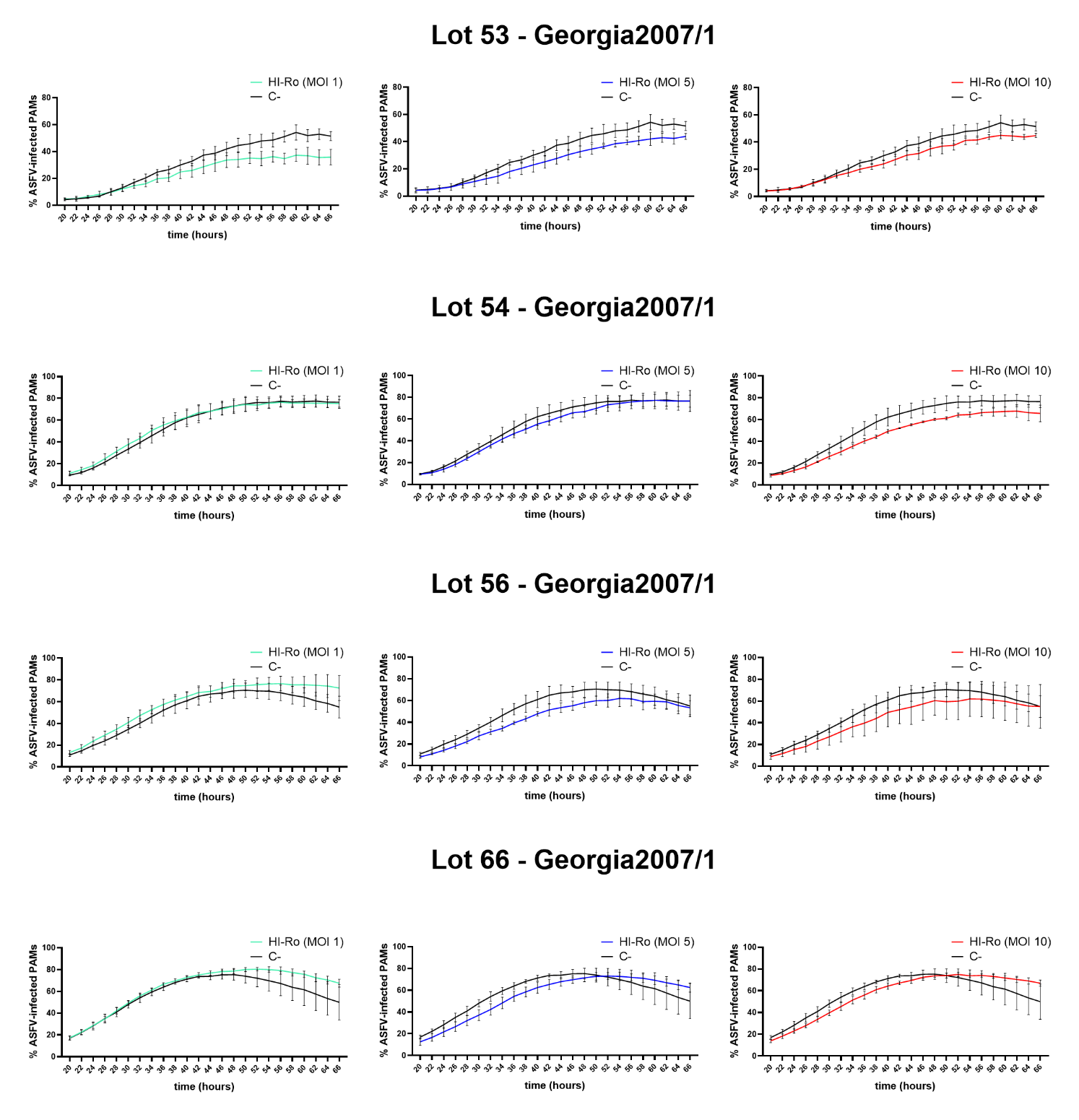
**

**Figure S7. Percentages of Georgia2007/1 fluorescent-labelled infected cells analyzed every 2 hours every individual porcine alveolar macrophage lot.** Data coming from 3 experimental replicates grouped by PAM lot (53, 54, 56, and 66) and treatment concentration (HI-RO MOI 1, 5, and 10). Non-infected cells were used as negative control. Values represent the mean ± SD for each condition.


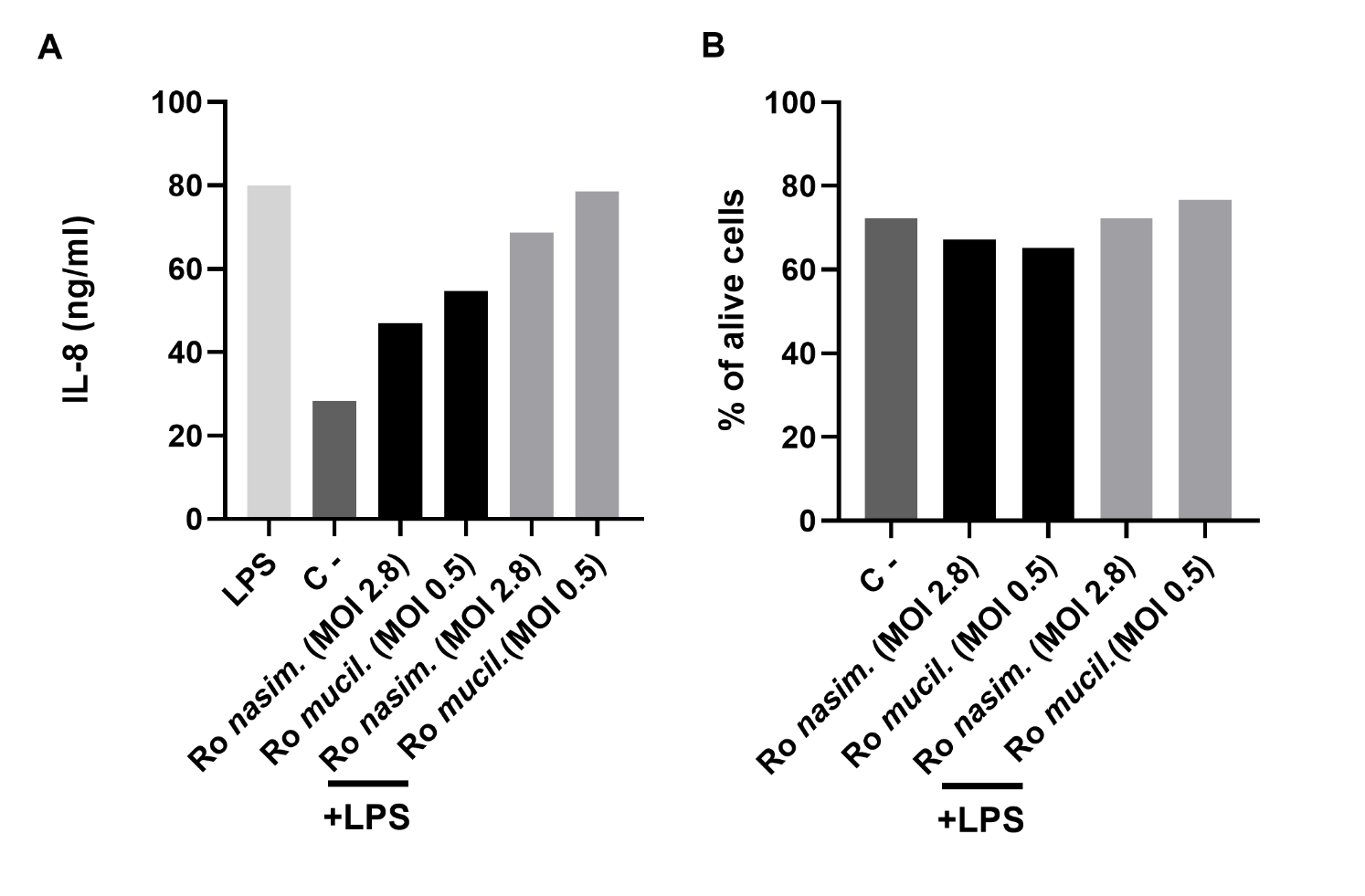


**Figure S8. *Rothia nasimurium* does not suppress the production of IL-8 in porcine alveolar macrophages.** (**A-B**) PAMs were stimulated for 24 hours with LPS (100 ug/mL) alone or in combination with alive *Rothia nasimurium* (Ro nasim.) or *Rothia mucilaginos*a (Ro mucil.) at the indicated MOIs. Non-stimulated cells were used as negative control (C-). (**A**) Level of IL-8 in cell supernatants was quantified by ELISA at 24 hours post-stimulation. (**B**) Percentages of alive cells were measured by flow cytometry using DAPI stain kit.
